# Modern alongside traditional taxonomy—Integrative systematics of the genera *Gymnangium* Hincks, 1874 and *Taxella* Allman, 1874 (Hydrozoa, Aglaopheniidae)

**DOI:** 10.1371/journal.pone.0174244

**Published:** 2017-04-19

**Authors:** Marta Ronowicz, Emilie Boissin, Bautisse Postaire, Chloé Annie-France Bourmaud, Nicole Gravier-Bonnet, Peter Schuchert

**Affiliations:** 1 Department of Marine Ecology, Institute of Oceanology Polish Academy of Sciences, Sopot, Poland; 2 USR3278 Centre de Recherche Insulaire et Observatoire de l'environnement, Université de Perpignan, Perpignan, France; 3 Aix Marseille Université, CNRS, IRD, Avignon Université, IMBE UMR 7263, Marseille, France; 4 Université de La Réunion, UMR ENTROPIE, Faculté des Sciences et Technologies, Saint Denis, France; 5 Laboratoire d’Excellence Corail, Perpignan, France; 6 Natural History Museum of Geneva, Geneva, Switzerland; Instituto Español de Oceanografía, SPAIN

## Abstract

We studied the diversity within the former genus *Gymnangium* in the South West Indian Ocean by using an integrative approach of both traditional (morphology-based) and modern molecular taxonomy. Nine species were recorded in the material collected. A total of 97 16S mitochondrial DNA sequences and 54 Calmodulin nuclear sequences from eight *Gymnangium/Taxella* species were analyzed. We found both morphological and molecular differences in the studied *Gymnangium* species that make it necessary to split the genus. It is proposed to revalidate the genus *Taxella* which is currently regarded as a synonym of *Gymnangium*. Two species of the genus *Taxella* (*T*. *eximia* and *T*. *gracilicaulis*), until now regarded as distinct species based on morphological characteristics, cluster together in one phylogenetic clade. Possible explanations are discussed. Two species from Madagascar new to science are herein described and rare species from the Indian Ocean islands are re-described.

## Introduction

*Gymnangium* Hincks, 1874 is a genus of the family Aglaopheniidae Marktanner-Turneretscher, 1890 with currently about 41 accepted nominal species (Table 1) [1]. The majority of the *Gymnangium* species occur in the tropical and subtropical waters of the Indian and Pacific Oceans. Only about five species have been documented reliably in Atlantic waters (*G*. *allmani* (Marktanner-Turneretscher, 1890), *G*. *montagui* Billard, 1912, *G*. *sinuosum* Fraser, 1925, *G*. *arcuatum* (Lamouroux, 1816), and *G*. *speciosum* Allman, 1877 [2–8]. A single one (*G*. *montagui*) has barely penetrated into the Mediterranean Sea [9].

**Table 1:** Morphological characteristics of genera *Taxella* and *Gymnangium* (species included in the molecular phylogeny are in bold).

	Clade on tree Figs 4 and 5	Polysi-phonic stem	Branched colony	Abcauline hydrothecal septum	Hydrotheca Stechow type "*Halicetta*"	Hydrotheca Stechow type "*Haliaria*"	Hydrotheca Stechow type "*Gymnangium*"	Referenc
***Taxella eximia* (Allman, 1874)**	**A**	yes	yes	no	yes	no	no	Rees & Vervoort 1987 [48], present study
***Taxella gracilicaulis* (Jäderholm, 1903)**	**A**	yes	yes	no	yes	no	no	Rees & Vervoort 1987 [48], present study
***Taxella hornelli* (Thornely, 1904)**	**A**	yes	yes	no	yes	no	no	Present study
***Gymnangium millardi* Ronowicz sp. nov**	**B**	no	no	yes	no	no	yes	Present study
**?*Gymnangium expansum* (Jäderholm, 1903)**	**B**	no	yes	no	yes	no	no	Vervoort 1966 [56], present study
***Gymnangium hians* (Busk, 1852)**	**B**	no	no	yes	no	no	yes	Millard 1975 [7], present study
***Gymnangium insigne* (Allman, 1874)**	**B**	no	no	yes	no	no	yes	Allman, 1876 [14]
***Gymnangium montagui* (Billard, 1912)**	**B**	no	no	yes	no	no	yes	Cornelius 1995 [5]
*Taxella elfica* Ronowicz sp. nov.	no data	yes	yes	no	yes	no	no	Present study
?*Taxella explorationis* Vervoort & Watson, 2003	no data	yes	yes	no (adcauline ledge)	yes	no	no	Vervoort & Watson, 2003 [57]
*Taxella longicornis* (Busk, 1852)	no data	yes	yes	no	yes	no	no	Watson, 2000 [58]
*Taxella tubulifera* (Bale, 1914)	no data	yes	yes	no	yes	no	no	Vervoort & Watson, 2003 [57]
*Gymnangium allmani* (Marktanner-Turneretscher, 1890)	?no data	no	no	yes	no	no	yes	Galea 2013 [6]
*Gymnangium africanum* (Millard, 1958)	no data	no	no	no	no	yes	no	Millard, 1975 [7]
*Gymnangium arcuatum* (Lamouroux, 1816)	no data	no	no	no	no	yes	no	Millard, 1975 [7]
*Gymnangium ascidioides* (Bale, 1882)	no data	no	no	yes	no	no	yes	Watson, 2005 [59]
*Gymnangium aureum* (Watson, 1973)	no data	no	yes	yes	no	no	yes?	Watson, 1973 [60]
*Gymnangium australimage* Watson, 2005	no data	no	no	yes	no	no	yes	Watson, 2005 [59]
*Gymnangium baileyi* (Bale, 1884)	no data	no	yes	yes	no	no	yes	Bale, 1884 [61]
*Gymnangium birostratum* (Bale, 1914)	no data	no	no data	no	in part (?)	no	in part (?)	Vervoort & Watson, 2003 [57]
*Gymnangium bryani* (Nutting, 1906)	no data	no	no	yes	no	no	yes	Present study
*Gymnangium comes* (Briggs, 1938)	no data	no	yes	yes	no	no	yes	Hodgson, 1950 [62]
*Gymnangium exsertum* (Millard, 1962)	no data	no	no	no	no	yes	no	Millard, 1975 [7]
*Gymnangium ferlusi* (Billard, 1901)	no data	no	no	no	no	yes	no	Present study
*Gymnangium furcatum* (Bale, 1884)	no data	no	yes	yes	no	no	yes	Bale, 1884 [61]
?*Gymnangium goniodes* (Briggs, 1915)	no data	no	yes and no	no	no	yes	no	Briggs, 1915 [63] (genus unclear = *Aglaophenia*?)
Gymnangium humile (Bale, 1884)	no data	no	no	no	no	yes	no	Vervoort & Watson, 2003 [57]
*Gymnangium ilicistomum* (Bale, 1882)	no data	no	no	no	no	yes	no	Watson, 2005 [59]
*Gymnangium indivisum* (Fraser, 1936)	no data	unknown	no	no	no	yes	no	Fraser, 1936 [64]; Yamada, 1959 [19]
*Gymnangium ishikawai* (Stechow, 1907)	no data	no	no	yes	no	no	yes	Stechow, 1907 [65]
*Gymnangium japonicum* Watson & Vervoort, 2001	no data	no	no	no	no	yes	no	Watson & Vervoort, 2001 [66]
*Gymnangium longirostre* (Kirchenpauer, 1872)	no data	no	yes	no	no	yes	no	Bale, 1884 [61]
*Gymnangium magnirostre* (Nutting, 1927)	no data	yes		no	no	yes	no	Nutting, 1927 [67]
*Gymnangium prolifer* (Bale, 1882)	no data	no	yes	no	no	yes	no	Watson, 2005 [59]
*Gymnangium roretzii* (Marktanner-Turneretscher, 1890)	no data	no	no	yes	no	no	yes	Schuchert, 2015 [68]
*Gymnangium sinosum* (Fraser, 1925)	no data	no	no	yes	no	no	yes	Calder, 1997 [3]
*Gymnangium speciosum* (Allman, 1877)	no data	no	no	yes	no	no	yes	Calder, 1997 [3]
*Gymnangium superbum* (Bale, 1882)	no data	no	no	yes	no	no	yes	Watson, 2005 [59]
*Gymnangium tenuirostre* (Nutting, 1927)	no data	no	no	yes	no	no	yes	Nutting, 1927 [67]
*Gymnangium thetidis* (Ritchie, 1911)	no data	no	no	no	no	yes	no	Ritchie, 1911 [69] (= *Aglaophenia*?)
*Gymnangium undulatum* Watson, 2000	no data	no	no	yes	no	no	yes	Watson, 2000 [58]
*Gymnangium urceoliferum* (Lamarck, 1816)	no data	no	no	no	no	yes	no	Billard, 1907b [70]
*Gymnangium vegae* (Jäderholm, 1903)	no data	yes	yes	no	no	yes	no	Jäderholm, 1903 [71]

Hincks [10] introduced the name *Gymnangium* for species previously included in the genus *Aglaophenia* Lamouroux, 1812 but bearing unprotected gonothecae on the stem. Stechow [11] later selected *Halicornaria montagui* Billard, 1912 as the type species of the genus. The remaining genera of the Aglaopheniidae have gonothecae protected by accessory structures, e.g. modified hydrocladia developing into corbulae (as for *Aglaophenia* Lamouroux, 1812 and *Lytocarpia* Kirchenpauer, 1872) or to phylactocarps (as for *Cladocarpus* Allman, 1874 and *Macrorhynchia* Kirchenpauer, 1872) (for more details see Bouillon et al. [12]). Calder [3] created two subfamilies for these two different groups, the Gymnangiinae Calder, 1997 and the Aglaopheniinae Marktanner-Turneretscher, 1890, and he provided an extensive taxonomic history and synonymy for *Gymnangium*.

Allman [13] introduced the genus *Taxella* (type species *Taxella eximia* Allman, 1874, currently *G*. *eximium*), a name which was not used after Allman—practically a nomen nudum—likely because the type species was inadequately described and because *T*. *eximia* was later given another name by Allman [14], that is *Halicornaria bipinnata*. For many years there was another generic name, *Halicornaria* Allman, 1874, widely used as synonym for *Gymnangium*. However, *Halicornaria* Allman, 1874 is a junior homonym of *Halicornaria* Hincks, 1865 which was first used to denote a different taxon [3]. Therefore, the name *Gymnangium* is recognized as the valid name of the genus nowadays.

**Fig 1:**
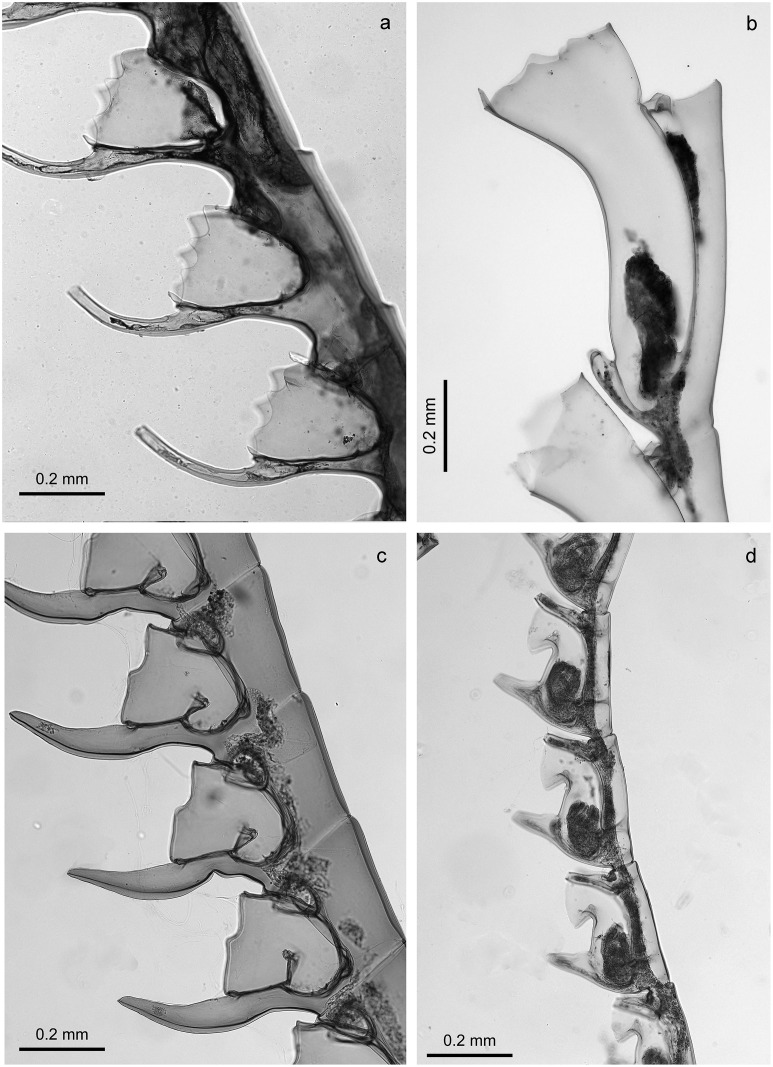
Hydrothecal forms in genera *Gymnangium*/*Tax*ella: a) *Gymnangium longirostre*, MHNG-INVE-79820, New Zealand ("*Haliaria*" form of Stechow), b) *Gymnangium expansum* MHNG-INVE-69623, Okinawa ("*Halicetta*" form of Stechow), c) *Gymnangium montagui*, MHNG-INVE-89694, Brittany ("*Gymnangium*" form of Stechow), d) *Taxella gracilicaulis*, MHNG-INVE-32462 (typical hydrothecal form of the genus *Taxella*).

Stechow [15] considered the genus *Gymnangium* problematic as it was based on the absence of a character, an astonishingly modern thought for his time. Although not a solution to this problem, Stechow split *Gymnangium* into three genera on the basis of hydrothecal morphology (Fig 1):

1. *Haliaria* Stechow, 1921 (type species *Halicornaria vegae* Jäderholm, 1903) with a sac-like hydrotheca, not bent or folded, and without an abcauline intrathecal septum (Fig 1a);
2. *Halicetta* Stechow, 1921 (type species *Halicornaria expansa* Jäderholm, 1903) with an elongated hydrotheca, slightly curving but not bent, without an abcauline intrathecal septum (Fig 1d);
3. *Gymnangium* with a sac-like hydrotheca, bent or folded, with a large abcauline intrathecal septum (type species *Halicornaria montagui* Billard, 1912 by designation of Stechow [3] (Fig 1c).

Leloup [16] did not deem these differences as sufficient enough to represent full genera and he demoted them to subgenera (which is taxonomically equivalent). Calder [3], following Bouillon [17] and Vervoort [18], advocated the use of the broader scope of *Gymnangium sensu* Hincks, until further insights on the relationships among species and on the nature of the gonophore were provided. Stechow’s subdivision of *Gymnangium* was therefore not used during the last few decades, except for Yamada [19] and Hirohito [20].

The identification and separation of the species within this genus can be difficult due to considerable morphological intraspecific variability [3]. A wide phenotypic plasticity is a well-known characteristic of hydroids [21]. Substantial variations in hydroid morphology have been reported in response to different environmental conditions (e.g., temperature, salinity, water movement) [22–23]. This feature often makes the taxonomists’ work complicated and hinders unambiguous identification to the species level. In recent years, a molecular tool has been developed and was purported to solve many of the species identification problems—DNA barcoding [24–25]. This method is evolving rapidly and it is becoming a very practical aid for the identification of species and populations, clarifying synonymy problems and providing new insights into evolution [26–30]. Applied to the Aglaopheniidae, the results of such DNA barcoding studies put in evidence both concordances [29] and discordances [31] between the traditional classification established on morphological characters and the phylogenetic trees. One such discordance was the strong genetic divergence observed in the examined *Gymnangium* species.

Using a combination of DNA sequence data and morphological characters we investigated the taxonomic identity of specimens belonging to several *Gymnangium* species collected mainly in the South West Indian Ocean (SWIO), around three of the French Scattered Islands (Glorieuses, Juan de Nova and Europa Islands), Geyser, Mayotte, Madagascar and Reunion Island, and in the Maldives Archipelago (northern part of the Indian Ocean). Our results confirmed the necessity to split the genus *Gymnangium* in at least two genera. Thus, we propose here to use the genus *Taxella* Allman, 1874 for some of the species studied.

To summarize, the aims of the present study were i) to use integrative methods of taxonomy (both morphological and DNA barcoding analyses) in order to describe diversity within the former genus *Gymnangium* of the Indian Ocean, ii) to re-describe some poorly known *Gymnangium* species, and iii) to present a description of two new species from Madagascar, *Taxella elfica* and *Gymnangium millardi*.

## Study area

The study area, the SWIO, encompasses two marine biodiversity hotspots [32] as well as a recently identified ‘core region’ of biodiversity at the north Mozambique Channel [33–34]. This area was also recently proposed as an evolutionary hotspot, a region able to both generate and maintain biodiversity over a long period of time [35]. The scattered Islands in the Indian Ocean constitute a diverse and unique habitat for numerous marine species and present a high hydroid species richness [36–42].

The major current supplying the SWIO originates from the East part of the basin: the South Equatorial Current flowing from the biodiversity golden triangle hot spot and splitting when it approaches the Malagasy eastern coast [43]. The northern flow diverges close to the Comoros with one part running southwards into the Mozambique Channel in a series of dynamic cyclonic and anticyclonic eddies propagating southwards along the western edge of the Channel [44] nourishing finally the Agulhas Current along the East coast of South Africa, and a second part flowing northwards as the East African Coastal Current, supplying the Somali Current.

**Fig 2:**
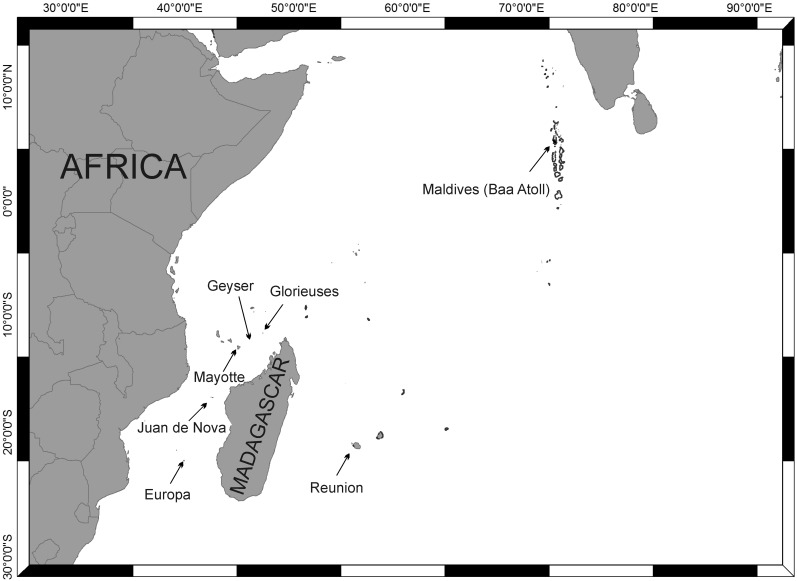
Map of the study area.

Hydroids were collected around five islands (or coral banks) located in the Mozambique Channel between Africa and Madagascar (Glorieuses, Geyser, Mayotte, Juan de Nova and Europa), and from Madagascar and Réunion Island (Fig 2). Some colonies were also collected in Baa Atoll of the Maldives Archipelago in the North of the Indian Ocean (Fig 2).

## Methods

"None of the species investigated were endangered or protected. Collection permits were provided by local authorities as follows: Direction de l'Agriculture et de la Foret (for Mayotte), Terres Australes et Antarctiques Françaises (for Scattered Islands), Affaires Maritimes (for Reunion Island), Ministry of Fisheries and Agriculture (for the Maldives)".

**Fig 3:**
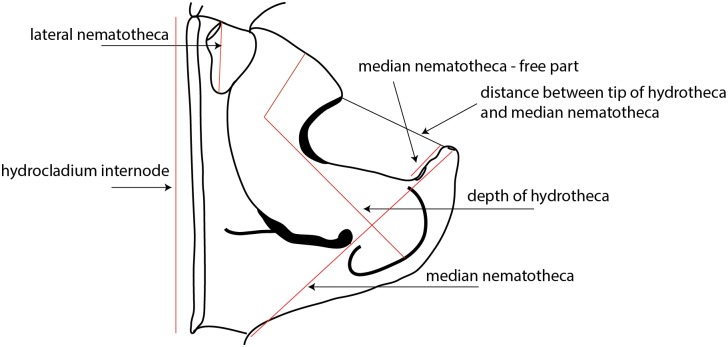
Measurements methodology.

The hydroid samples were collected in shallow waters by scuba diving or snorkeling during several marine biodiversity field trips to the SWIO islands from 2004 to 2013. The material was sorted alive and preserved in formalin. In addition, subsamples were preserved in 95% ethanol for subsequent DNA analyses. Some older samples of interesting species from the same region preserved in formalin and microscopic slides (from the collection of the Natural History Museum of Geneva (MHNG) and private collection of Nicole Gravier-Bonnet & Chloé Bourmaud—NGB&CB) were also studied morphologically. Unfortunately, they could not be used in molecular analyses. Hydroids were identified to the morphospecies level using the taxonomic literature, among them several monographic studies [7,37,45–48]. Drawings were prepared using a microscope combined with a *camera lucida* and redrawn on a digital tablet as proposed by Coleman [49]. The particular dimensions were measured with the use of Leica Application Suite (Fig 3). When more than three specimens were measured, mean and standard deviation are given. Otherwise, a range (minimum—maximum) is provided.

Principal Components Analysis (PCA) was applied using PRIMER 6 [50] to evaluate systematic relationships based on morphological characters among nine nominal species attributed to *Gymnangium* and *Taxella*. The data matrix employed consisted of 25 characters given for 86 specimens (see S3 Appendix). The data was column-standardized prior to the analysis as recommended by Thorpe [51].

### Nomenclature acts

The electronic edition of this article conforms to the requirements of the amended International Code of Zoological Nomenclature, and hence the new names contained herein are available under that Code from the electronic edition of this article. This published work and the nomenclatural acts it contains have been registered in ZooBank, the online registration system for the ICZN. The ZooBank LSIDs (Life Science Identifiers) can be resolved and the associated information viewed through any standard web browser by appending the LSID to the prefix “http://zoobank.org/”. The LSID for this publication is:

urn:lsid:zoobank.org:pub:4AD7F25F-D850-44FA-A67F-EEB822401FBC. The electronic edition of this work was published in a journal with an ISSN, and has been archived and is available from the following digital repositories: PubMed Central, LOCKSS.

### Molecular analyses

DNA was extracted from hydranths using the DNeasy kit (Qiagen, Hilden, Germany). A portion of the mitochondrial 16S gene was amplified using the primers 16S-SHA and 16S-SHB [52]. PCR mixture and cycling parameters were as given in this publication. PCR products were sent for sequencing to a private company (GenoScreen, Lille, France). A total of 97 sequences of 7 *Gymnangium/Taxella* species from the Indo-Pacific region were included in the analyses (see S1 Appendix for GenBank accession numbers). Additionally, sequences of *G*. *montagui* from the Atlantic Ocean were available from GenBank (JN560075-JN560078) and included in the analyses. *Gymnangium insigne* specimens from Tahiti used for phylogenetic analysis were identified by Gravier-Bonnet and the 16S sequences were published by Postaire et al. [31]. Additionally sequences of *G*. *expansum* from Japan were obtained for this study.

Specimens from other Aglaopheniidae from the South West Indian Ocean region (*Lytocarpia nigra* (Nutting, 1906), *L*. *brevirostris* (Busk, 1852), *Macrorhynchia phoenicea* (Busk, 1852), *Aglaophenia cupressina* Lamouroux, 1816) were also sequenced and used in phylogenetic reconstruction (GenBank numbers: KU594426-KU594431; KU594433; KU594442).

Finally, following Moura et al. [29] five *Schizotricha* species available from GenBank were used as outgroup (GenBank accession numbers: FN424125-FN424129).

Additionally, a portion of the nuclear gene Calmodulin was amplified using the primers Cam-F1 and Cam-R1 [53] and the PCR conditions and cycling parameters as given in this publication.

### Phylogenetic analyses

Sequences were aligned online using MAFFT [54]. Maximum Likelihood reconstructions of phylogenetic relationships among species were performed using PhyML online [55]. A bootstrapping procedure was applied to assess support of the nodes (100 bootstraps).

## Results

Nine species of the former genus *Gymnangium* were recorded in the material collected in the study area (*Taxella eximia*, *T*. *gracilicaulis*, *T*. *hornelli*, *T*. *longicornis*, *T*. *elfica* sp. nov., *Gymnangium hians*, *G*. *bryani*, *G*. *millardi* sp. nov., *G*. *ferlusi*). Altogether 259 specimens were examined morphologically. A total of 97 mitochondrial 16S sequences and 54 nuclear Calmodulin sequences of *Gymnangium/Taxella* species were analyzed. Four species (*T*. *longicornis*, *T*. *elfica* sp. nov., *G*. *bryani*, *G*. *ferlusi*) were not included in molecular analyses due to a lack of sequence data.

We found morphological and molecular differences in studied *Gymnangium* species that make it necessary to split the genus. It is proposed to revalidate the genus *Taxella* which is currently regarded as a synonym of *Gymnangium*.

### Phylogenetic analysis

**Fig 4:**
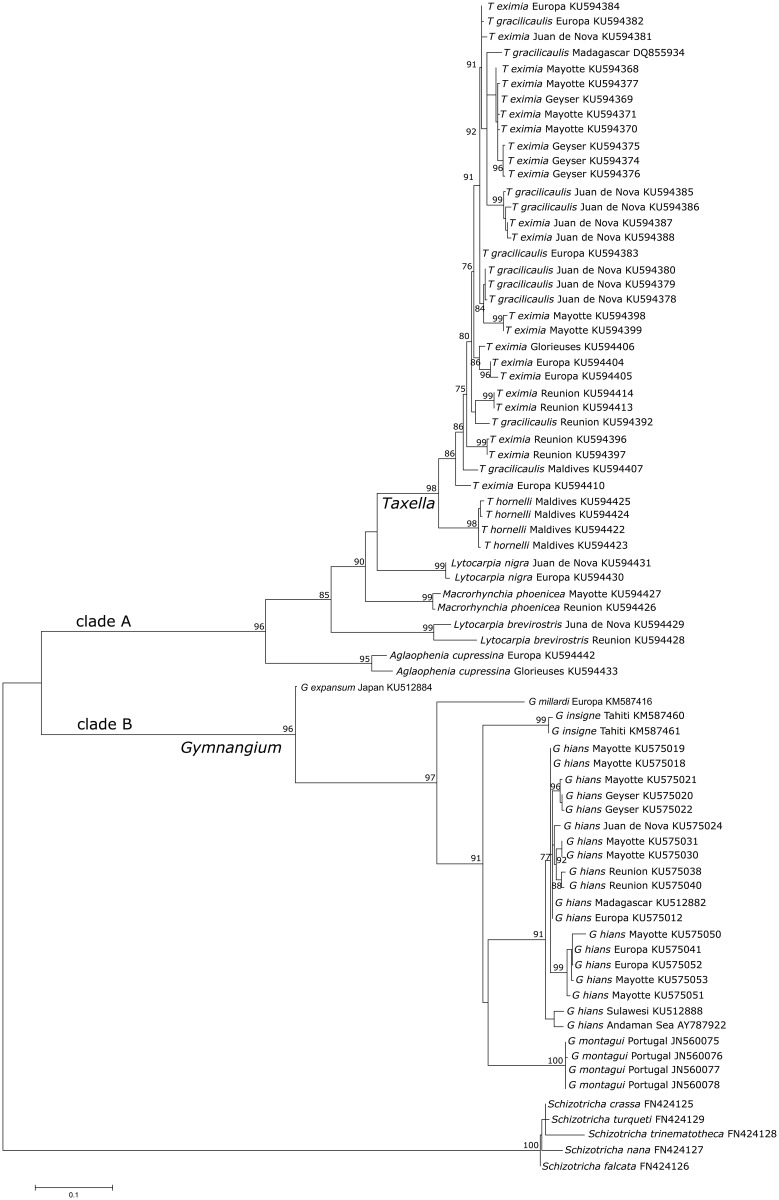
Maximum likelihood reconstruction of the phylogenetic relationships among species of the *Gymnangium* and *Taxella* genera using the 16S mitochondrial gene. Numbers at nodes are bootstrap values. *Schizotricha* species are used as outgroups.

**Fig 5:**
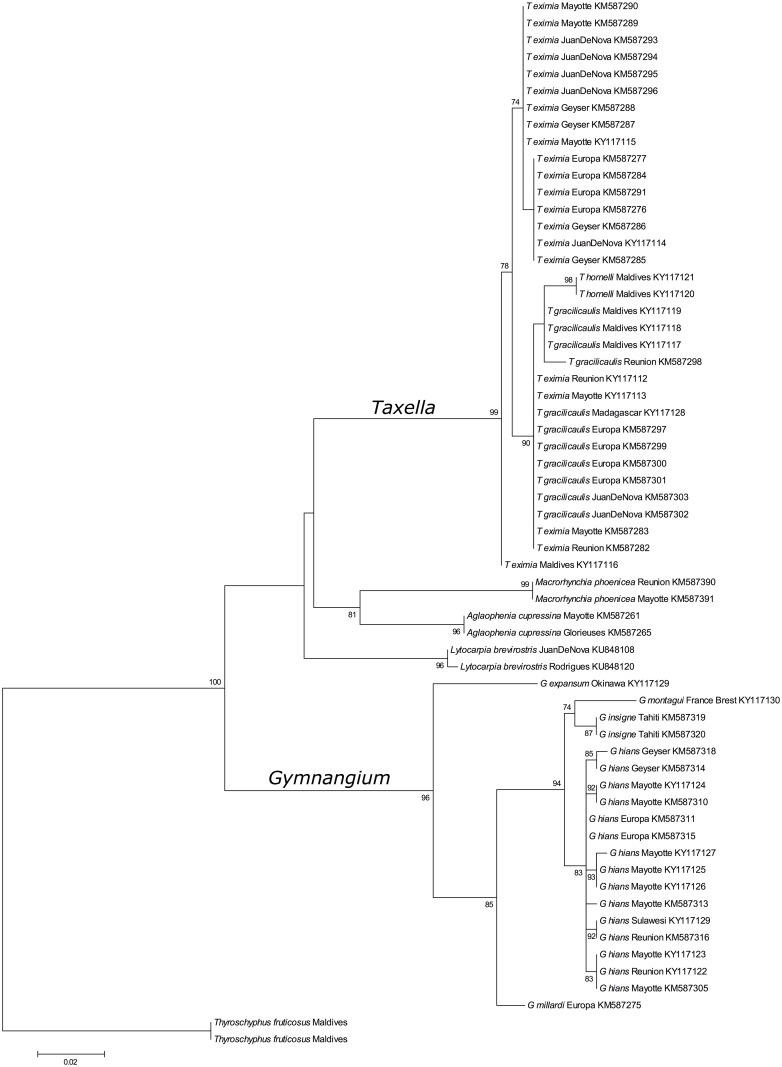
Maximum likelihood reconstruction of the phylogenetic relationships among species of the *Gymnangium* and *Taxella* genera using the Calmodulin gene. Numbers at nodes are bootstrap values. *Schizotricha* species are used as outgroups.

Phylogenetic reconstructions of the *Gymnangium* species analyzed in this study are presented on Figs 4 and 5. Species of *Gymnangium* form two well-supported clades (bootstrap value 96). On the phylogenetic tree constructed with the mitochondrial gene, Clade A comprises *Taxella* spp. (bootstrap value 98) which cluster with other Aglaopheniidae of the genera *Macrorhynchia*, *Aglaophenia* and *Lytocarpia*. As expected from Postaire [31,33], the two *Lytocarpia* species (*L*. *nigra* (Nutting, 1906) and *L*. *brevirostris* (Busk, 1852)) do not cluster together.

Within Clade A, a first cluster gathers specimens belonging to *T*. *hornelli* (bootstrap value 98). The second cluster contains both *T*. *eximia* and *T*. *gracilicaulis* with a support value of 86. Within *Gymnangium*—Clade B, three groups are distinguishable. A first split sets apart *G*. *expansum*, the second one *G*. *millardi* sp. nov. A third group contains *G*. *montagui* (from the Atlantic), *G*. *insigne* (collected in French Polynesia) and *G*. *hians*.

The phylogenetic tree based on the nuclear gene also segregates the species into two distinct clades, one grouping *T*. *gracilicaulis*, *T*. *eximia* and *T*. *hornelli* (bootstrap value 99) and another one grouping *G*. *hians*, *G*. *expansum*, *G*. *insigne*, *G*. *millardi* (bootstrap value 96). The relationships of the *Aglaophenia* and *Macrorhynchia* species, however, remain unresolved as the clade support values are not sufficiently high enough.

Thus, both phylogenetic reconstructions, with the nuclear and the mitochondrial gene, support a split of the genus *Gymnangium* in two distinct groups.

### Morphological analysis

**Fig 6:**
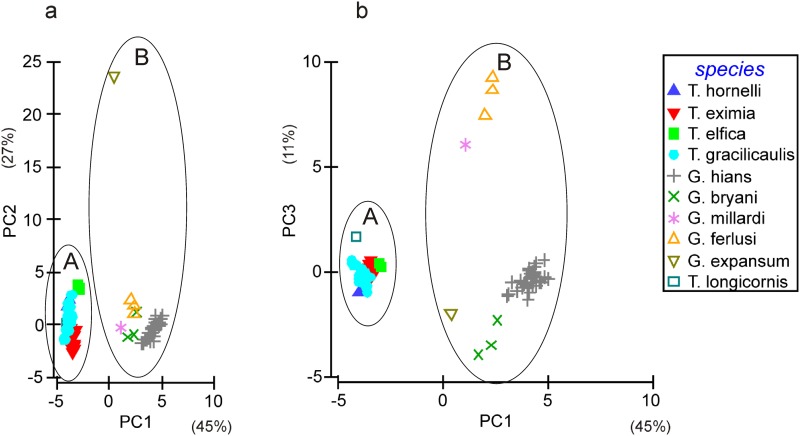
PCA diagrams based on 25 morphological characters for the first three axes: a) PC1 and PC2, b) PC1 and PC3.

The two *Gymnangium* clades identified by phylogenetic reconstruction possess diagnostic characters, allowing morphological distinction. The systematic relationships between the species under study were assessed based on 25 morphological characters using a Principal Components Analysis (PCA) (Fig 6a and 6b, for the list of applied morphological characters see S2 Appendix). Nearly 92% of the inter-specific variability was explained by the selected morphological parameters used in the PCA. PC1 accounted for 45% of the total variation in the data and separated specimens into two groups, supporting the division within the genus *Gymnangium*. This axis was associated with the characters pertaining to stem appearance (presence of polysiphonic stem and axial tube with short hydrocladia), morphometry of hydrocladia, hydrothecal shape, number of marginal teeth and diameter at mouth, presence of abcauline intrathecal septum, median nematotheca shape and its terminal aperture end (for contribution of each character see S3 Appendix). PC2 accounted for 27% of variance and was mainly associated with size variables of hydrotheca depth, distance between tip of median nematotheca and abcauline tip of hydrotheca and hydrocladia internode length. PC2 splitted up *G*. *expansum* from the rest of specimens due to much larger size of hydrothecae and longer hydrocladial internodes. This axis also revealed morphometric intra-specific variability within single species.

The separation between *Gymnangium*/*Taxella* species is more evident when drawn in the PC1 versus PC3 plot (Fig 6b). PC3 explained 11% of variance and did not create a long gradient as in the case of PC2. PC3 was associated with length of lateral nematothecae and median nematothecae, length of a free part of median nematothecae and number of apertures of median nematothecae.

The considerable overlap among different species within *Gymnangium* and *Taxella* group respectively disappeared when the analysis was conducted for both genera separately (not shown).

The cluster including *Taxella eximia* (*Gymnangium eximium* is the type species of *Taxella*), comprises species which have a polysiphonic stem, with an axial tube in front of stem from which the stem hydrocladia arise alternately and accessory tubes from which arise secondary branches (Table 1). The *Taxella* species included in the molecular analysis also have the same type of hydrotheca (Fig 1d), which resembles the *Halicetta* type but with a more curved distal end (Fig 1b–1d). However, the type species of *Halicetta* (*G*. *expansum*) belongs to Clade B and it has a monosiphonic stem. So, with our current knowledge, *Taxella* is characterized only by the combination of a polysiphonic stem and *Halicetta*-like hydrotheca.

Most species of the genus *Gymnangium* have a monosiphonic stem (Table 1). The majority of species share other common features i.e. feather like colony form, cup-shaped hydrothecae, which is bent or folded, with or without an abcauline intrathecal septum (hydrotheca type *Haliaria*). Interestingly, *G*. *expansum* which clustered with *Gymnangium* clade has some intermediate morphological features of both genera i.e. monosiphonic stem (similar to other *Gymnangium*) but elongated hydrotheca, not bent or folded, without abcauline intrathecal septum (resembling *Taxella*). Because the present 16S data as well as an earlier analysis of Postaire et al. [33] clearly indicate a polyphyly of the genus *Gymnangium* sensu Bouillon et al. [4] we prefer to separate the *Gymnangium* species of Clade A into a different genus, and for which there is conveniently already a name available, *Taxella* Allman, 1874.

## Discussion

Phylogenetic relationships of the family Aglaopheniidae (Marktanner-Turneretscher, 1890) (the most diverse family in the class Hydrozoa) have recently been studied by Moura et al. [29] and Postaire et al. [31]. Both studies found that the traditional subdivision of the family based on the morphology of the reproductive structures did only partially match the results of a molecular phylogenetic approach. Notably, both studies and the present one found that the genus *Gymnangium* is likely polyphyletic. The genus *Gymnangium* was traditionally diagnosed by the absence of protective structures associated with the gonothecae. However, the study by Moura et al. [29] and the present which used additionally also nuclear marker, grouped the *Gymnangium* species into two, well separated clades. The clade containing *G*. *hians*, *G*. *millardi*, *G*. *insigne*, *G*. *montagui* and *G*. *expansum* (Clade B on Figs 4 and 5) is separated from the second clade grouping together *T*. *gracilicaulis*, *T*. *eximia* and *T*. *hornelli*, and species of the genera *Macrorhynchia*, *Lytocarpia* and *Aglaophenia* (Clade A on Figs 4 and 5). For the latter *Gymnangium* species that clusters together with other genera, Postaire et al. [31] suggested to use the name *Halicetta* Stechow, 1921, although the type species of the genus was not included in the analysis. In the present study, it has been possible to include also the type species of *Halicetta*, *Halicornaria expansa* Jäderholm, 1903. Surprisingly, this species did not cluster with Clade A containing *T*. *eximia*, but with Clade B containing the type species of *Gymnangium*, *G*. *montagui*. The Clade A could not have been named *Halicetta* anyway, as an older genus name was available, namely *Taxella* Allman, 1874 which is linked to one of the species of Clade A, *Taxella eximia*.

Postaire et al. [31] raised the question about what shared trait could support the clade composed of the genera *Lytocarpia*, *Macrorhynchia* and *Taxella*. The answer could be the structure of the stem which is polysiphonic and composed of a primary tube from which stem hydrocladia arise and accessory tubes from which branches arise.

The separation of *Gymnangium* into two genera is confirmed by both molecular (16S and Calmodulin) and morphological analyses (PCA). *Gymnangium* incorporates species of Clade B on phylogenetic tree and group B on PCA diagrams (*G*. *hians*, *G*. *bryani*, *G*. *millardi*, *G*. *ferlusi*, *G*. *expansum*). *Taxella* includes species of Clade A on phylogenetic tree and group A on PCA diagram: *T*. *hornelli*, *T*. *eximia*, *T*. *gracilicaulis*, *T*. *longicornis* and *T*. *elfica*. Because *T*. *longicornis* and *T*. *elfica* have both also polysiphonic stems and a hydrotheca resembling the one of *T*. *eximia*, these species are here placed in the genus *Taxella*, although no 16S data could be obtained for these species.

Specimens identified morphologically as *Taxella eximia* and *Taxella gracilicaulis* cluster together in one clade but with no apparent separation into independent lineages (Figs 4 and 5). This may have several explanations: 1) they are actually conspecific, forming a phylogenetic species, 2) the two species are hybridizing, and 3) their speciation is so recent that both species still share the ancestral polymorphism of their common ancestor, viz. an incomplete lineage sorting. The two morphotypes are also sometimes difficult to distinguish (more details are given in the taxonomic part). Highly variable nuclear markers such as microsatellites are more promising to resolve this problem. This situation appears to be more common in hydroids as we observed a similar situation for two species of fire corals (*Millepora tenera* Boschma, 1949 and *Millepora dichotoma* (Forsskål, 1775)) where the 16S, Calmodulin or ITS genes show tangled haplotype and allele networks whereas microsatellite data clearly distinguish the two species (Boissin unpublished). A likewise complex situation was also found in another marine thecate hydroid, *Plumularia setacea* [72].

*Gymnangium* specimens of species with a hydrotheca conforming with Stechow's genus *Haliaria* could unfortunately not be obtained for this analysis. They are certainly needed for a comprehensive evaluation of the genus *Gymnangium* and it is hoped to obtain some during future surveys.

The present results show again that molecular and traditional taxonomy are not always straightforward with each other. Morphological characters used for discriminating morpho-species and higher taxa (in our case genera) may often not represent monophyletic groups. Due to the morphological simplicity and plasticity of hydrozoans, species limits are sometimes extremely blurred (e.g. as shown here for *T*. *gracilicaulis* and *T*. *eximia*). Phylogenetic studies often revealed that some useful morphological characters used to discriminate species or higher taxa joined specimens or species into unrelated groups thus providing evidence of discrepancy in traditional systematics. For the Aglaopheniidae, the presence of different structures protecting gonothecae or their absence was traditionally used to subdivide the family into genera. However, molecular data indicate that these structures have likely evolved several times independently (homoplasy) e.g. the corbulae in *Aglaophenia* [29,31] and *Lytocarpia* [31]. The situation for *Gymnangium* is somewhat different, as was already noted by Stechow [15]. The absence of protective structure is a non-derived state, a plesiomorphy, which becomes evident through comparisons with outgroups, viz. other members of the superfamily Plumularioidea. As the plesiomorphic state of a character does not permit to discover a common ancestor, it is in fact not so surprising that the genus *Gymnangium* turned out to be polyphyletic.

In conclusion, we would like to emphasize the power of an integrative approach in which classical and molecular taxonomy complement each other, and both contribute to a more accurate taxonomic valuation. Such a comprehensive approach allows to grasp and understand the relationship between species and higher taxa.

Both the nuclear and mitochondrial phylogenetic reconstructions and PCA based on morphological characters demonstrate the need to split the genus *Gymnangium* and resurrect *Taxella* to separate nominal genus of different evolutionary traits.

## Taxonomic part

### Genus *Taxella* Allman, 1874 emended

**Diagnosis**: Aglaopheniidae with unprotected gonothecae, polysiphonic stem, and elongated hydrotheca, with a curved away opening region, but not sharply bent and without an abcauline intrathecal septum. Gonothecae laterally compressed, having an elliptic section. Type species by monotypy *Taxella eximia* Allman, 1874.

### *Taxella eximia* (Allman, 1874)

Figs 7a and 8a–8d

**Fig 7:**
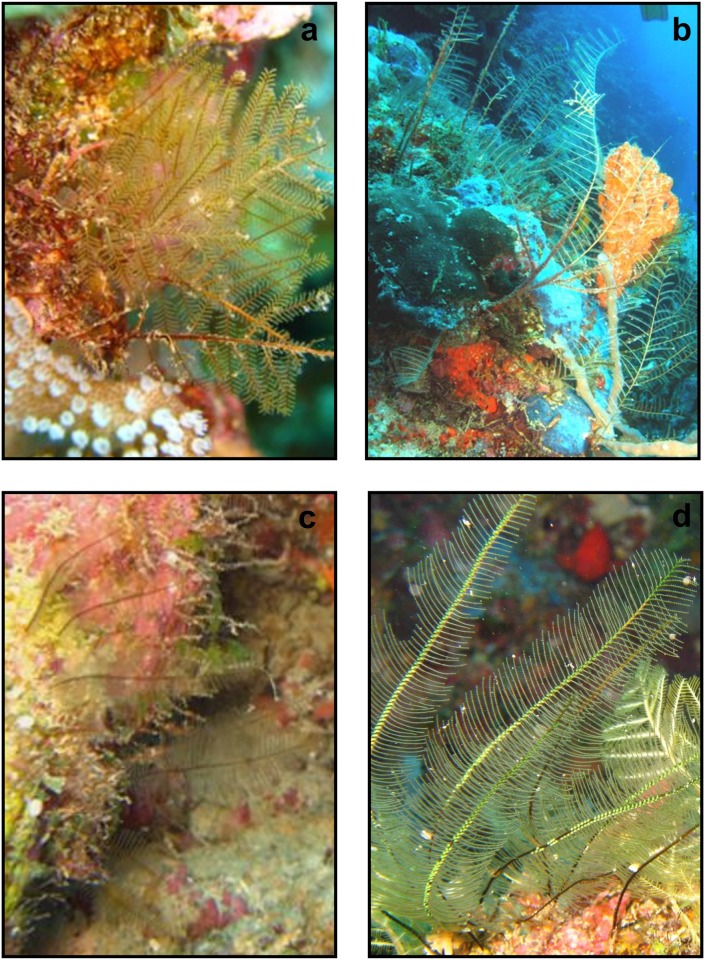
Underwater pictures from Mayotte: a) *T*. *eximia*, b) *T*. *gracilicaulis*, c) *G*. *hians*, d) *G*. *hians* fertile colonies (photos by Hendrik Sauvignet and Julien Wickel).

**Fig 8:**
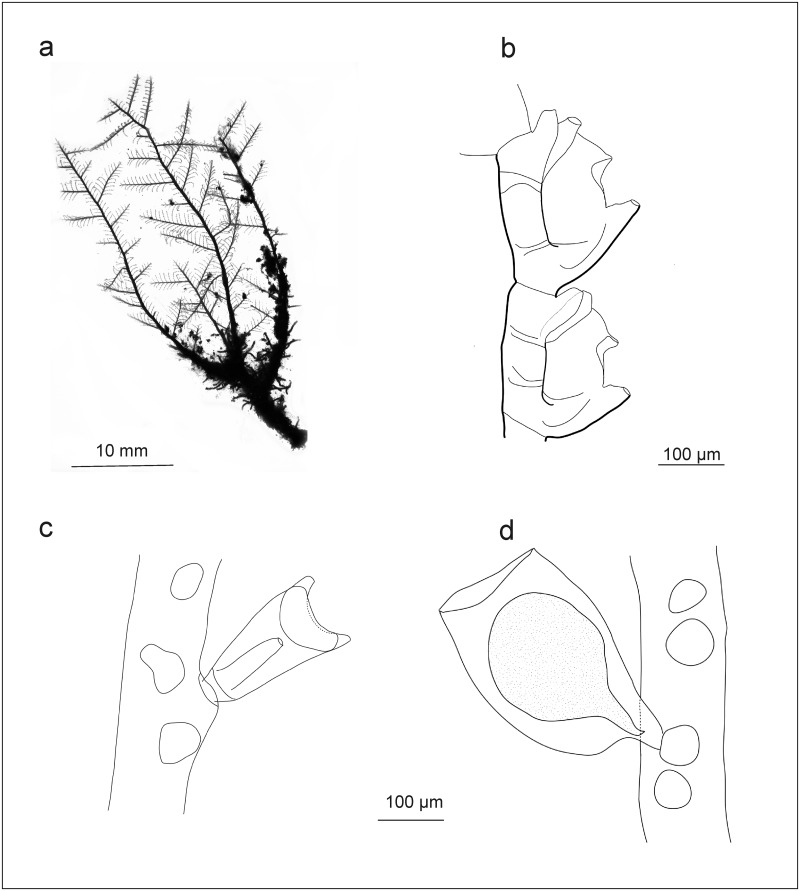
*Taxella eximia*: a) colony, b) part of hydrocladium, c) hydrotheca—frontal view, d) gonotheca.

*Taxella eximia* Allman, 1874: 179 [13].

*Taxella eximis*.- Kirchenpauer, 1876: 26 [73].

?*Halicornaria bipinnata*.- Allman, 1876: 279–280, pl. 22: fig. 5, pl. 23: fig. 2 [14]; Armstrong, 1879: 100 [74]; Schneider, 1897: 549 [75]; Bedot, 1921: 346 [76]; Von Schenck, 1965: 942 [77].

*Halicornaria setosa*.- Armstrong, 1879: 99–100, pl. 10 [74]; Thornely, 1904: 122 [78]; Bedot, 1921: 347 [76].

*Halicornaria flabellata*.- Marktanner-Turneretscher, 1890: 278–279, pl. 6: fig. 14 [79]; Schneider, 1897: 549 [75]; Bedot, 1921: 346 [76].

*Halicornaria gracilicaulis*.- in part Billard, 1907: 364, text-fig. 12, pl. 25: fig. 7 [37]; Millard, 1958:219, fig. 15 I,J [80].

*Halicornaria copiosa*.- Jarvis, 1922: 356, text-fig. 6, pl. 26: fig. 28 [81].

*Gymnangium eximium*.- Mammen, 1965: 311–312, fig. 104 [82]; Schmidt, 1972: 39,41,43, pl. 2: fig. C [83]; Mergner & Wedler, 1977: 22,24, pl. 6: fig. 40, pl. 9: figs. 62, 63 [84]; Vervoort & Vasseur, 1977: 81–84, fig. 34 [85]; Rees & Vervoort, 1987: 156–162, figs. 36, 37 [48]; Ryland & Gibbons, 1991 in part: 539–542, figs. 10,11,12 (not 11—*H*. *gracilicaule*) [86]; Vervoort, 1993: 549–550 [87]; El Beshbeeshy, 1995: 348–350, fig. 11B [88]; Kirkendale & Calder, 2003: 169 [89]; Gravier-Bonnet & Bourmaud, 2012: 105–106 and 113, fig. page 121 [40].

in part *Gymnangium eximium*.—Ryland & Gibbons, 1991: 539–542, figs. 10,12 (not 11 = *H*. *gracilicaule*) [86].

*Halicornaria gracilicaulis*.- Millard, 1958: 219, fig. 15I-J [80]; Vervoort, 1967: 47–50, figs. 14–15 [90].

*Halicornaria gracilicaulis lignose*.- Millard, 1968: 282–283 [91].

*Gymnangium gracilicaule lignosum*.- Millard, 1975: 443, fig. 136B-C, E [7].

#### Material examined

Totally 61 colonies inspected (for sample coordinates, depths, collection numbers see S1 Appendix).

Molecular data available (see S1 Appendix for GenBank accession numbers).

Type locality: Ceylon.

Distribution: Indian Ocean: Sri Lanka [14,82], off Kenya and Zanzibar [48], Seychelles [81], Arabian Sea [48], Red Sea [48,79,83,87,90]; Pacific Ocean: French Polynesia [85], Guam (Micronesia) [89], Fiji [86].

Depth: 0–2926 m, present material: between 0–40 m.

#### Description

Colour in situ: skeleton bright, red-brown for stem and branches, yellow-brown for hydrocladia, translucent for hydrocladia; coenosarc white, hydranth tentacles transparent.

Hydrorhiza root-like for attachment to hard substrata.

Colony multipinnate, usually rigid and strictly in a plane, reaching a height of up to 7 cm, branched, with a frontal and a posterior side defined by the location and orientation of the hydrocladia (Fig 8a, for underwater picture see Fig 7a).

Stem polysiphonic, with primary axial tube on the frontal side bearing alternate hydrocladia and a row of nematothecae, one basal and one axillary to each hydrocladium.

Branches polysiphonic, up to 4.5 cm long, springing alternately from the accessory tubules, with no clearly demarcated apophyses. Branches divided into internodes, separated by oblique nodes, bearing slightly curved hydrocladia in a plane. Secondary ramifications observed in the present material. Branchlets (the last-order branches) monosiphonic arising from the accessory tubules without a demarcated apophysis and with no hinge-joint. Three to five nematothecae at the basal part of each branchlet, below first hydrocladium.

Hydrocladia segmented homogenously by distinct nodes, each node with a hydrotheca, hydrothecae not overlapping. Internodes with two thickenings in the form of rings, one proximal, linked to the lateral thickenings of the hydrotheca, and the second one distal, at the level of insertion of the lateral nematothecae. Hydrocladia arising from branches, up to 2 mm, bearing 3 to 9 internodes, slightly curved and in a plane. Hydrocladia arising from the stem shorter, with 5 internodes at maximum, sometimes curved and encircling the stem, and with the most distal hydrothecae provided with lateral nematothecae that are longer than the others.

Hydrothecal axis S-shaped (Fig 8b), relatively short, rim even or slightly undulating but with no lateral tooth, making an angle of about 45° with the hydrocladium, abcauline wall nearly flat and parallel to the hydrocladium with upper part erected and bent to abcauline side, one straight adcauline thickening on the side of the hydrotheca arising from the level of the proximal internodal ring and reaching half of the lateral side or more. Distance between the distal end of the median nematotheca and the margin of the hydrotheca relatively short.

Median nematotheca perpendicular or oblique to internode, or sometimes slightly curved outwards, adnate to abcauline thecal wall for about half of its length, distal part free, two external apertures, one terminal circular, one adcauline at base of the free part, often with a shallow rim-like elevation, a third aperture connects the median nematotheca with the hydrothecal cavity at the upper end of the adnate part and a fourth one connects the nematophore with the coenosarcs inside the internode.

Lateral nematotheca tubular, upper end slightly narrowing, extending far over the level of hydrothecal aperture, facing upwards, one opening on top.

Gonothecae attached on the hydrocladium apophysis with a curved peduncle, flattened and either rounded (discoid) or with a truncated distal part, that can be provided with 2 short, horns-like lateral processes at distal end (Fig 8d). A central blastostyle surrounded by gametes forming a mass of coenosarc in the middle, itself surrounded by a ramification of the blastostyle. A single ovocyte per gonotheca.

Hydranth provided of 10 tentacles of unequal length.

Nematocysts: long and thin microbasic mastigophores gathered in bundles inside the lateral and median nematothecae.

Measurements given in S4 Appendix.

#### Remarks

We did not observe lateral cusps on the hydrothecal rim as did several authors [14,48,74,82–84,89,91]. In the present material, the margin is usually even, sometimes with a little undulation similar to that described by Marktanner-Turneretscher [79] for *Halicornaria flabellata* or drawn by Vervoort & Vasseur ([85] see p. 82, fig 34).

Some specimens from Fiji described by Ryland and Gibbons [86] as *G*. *eximium* (second form) are more likely *T*. *gracilicaulis* due to the delicate colony form, the elongated hydrothecae, the long and widely spaced branches. Their lumping of *G*. *gracilicaulis* into *G*. *eximium* was based on the incorrect assumption that the two species are distinguished by the presence of an abcauline septum in *G*. *eximium* ([86], p. 542, see under remarks). However, all the three forms of Ryland & Gibbons [86] were depicted without an abcauline intrathecal septum but with adcauline intrathecal ridge only ([86], figs 10,11,12), which is not a diagnostic character and may also be present in *G*. *gracilicaulis*.

*Halicornaria saccaria* Allman, 1876 was synonymised with *G*. *eximium* by Rees & Vervoort [48] but we suppose that it is probably a species of the genus *Macrorhynchia* due to the presence of modified hydrocladia protecting the gonotheca (phylactocarp).

*Taxella eximia* is sometimes difficult to differentiate from *T*. *gracilicaulis* (for a comparison see under remarks for *T*. *gracilicaulis*).

Some colonies are more delicate in the appearance having longer and more widely spaced hydrocladia. Not all the side-branches of a colony have the same orientation, some face the frontal side, others the posterior. The curvature at the abcauline side of the hydrotheca can be more or less pronounced. In this case, the only discriminating character was the absence of hinge-joints at the end of first segments of branches.

### *Taxella gracilicaulis* (Jäderholm, 1903) new comb.

Figs 7b and 9a–9d

**Fig 9:**
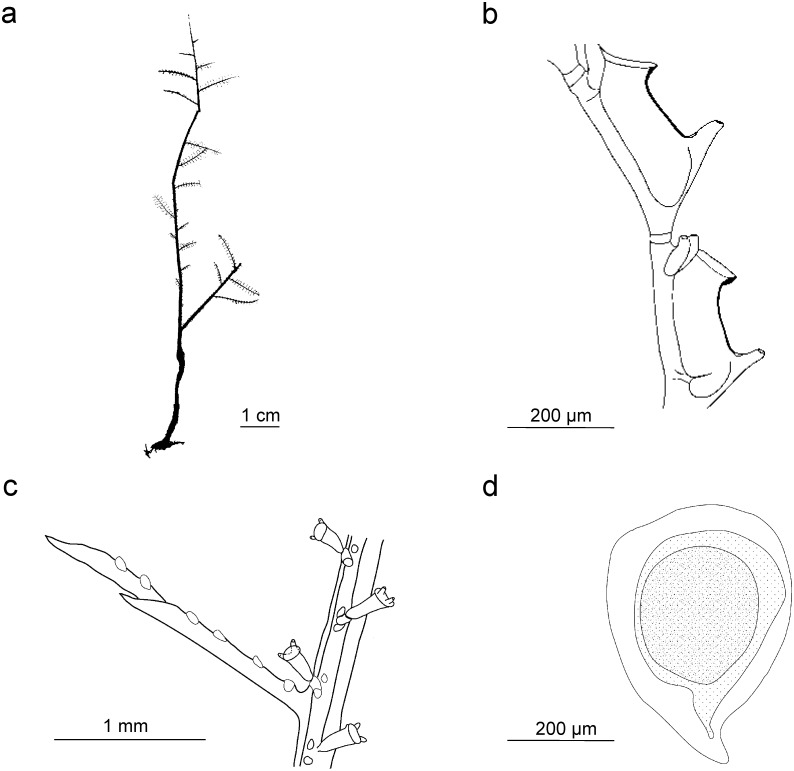
*Taxella gracilicaulis*: a) colony, b) part of hydrocladium, c) hinge joint and frontal view of stem, d) gonotheca.

*Lytocarpus gracilicaulis* Jäderholm, 1903: 299–300, pl. 14: figs. 3–4 [92].

Plumularide spec. (?) II Campenhausen, 1897: 316–317, figs. 2–3 [93].

*Halicornaria setosa*.- Thornely, 1904: 122 [78].

*Halicornaria gracilicaulis*.- Billard, 1907: 364–366, fig. 12, pl. 25: fig. 7 [37]; Ritchie, 1910: 23–24 [94]; Stechow, 1912: 368–369 [95]; Billard, 1913: 63 [45]; Jäderholm, 1919: 26 [96]; Jäderholm, 1920: 9–10, pl. 2: fig. 9 [97]; Bedot, 1921: 347 [76]; Billard, 1933:25, pl. 1: fig. 5 [98]; Dollfus, 1933:130 [99]; Von Schenck, 1965: 895, fig. 2d [77]; Millard, 1967: 191–192 [100].

(?) *Lytocarpus (*?*) hornelli*.- Thornely, 1908: 84 [101].

*Gymnangium (Halicetta) gracilicaulis*.- Stechow, 1921: 234 [15].

*Gymnangium gracilicaule*.- Rees & Thursfield, 1965: 170–171 [102]; Mammen, 1967: 310 [82]; Hirohito, 1983: 77 [103]; Rees & Vervoort, 1987: 168–172, fig. 40 [48]; Gravier-Bonnet & Bourmaud, 2012: 106 and 113, fig. page 121 [40]. *Gymnangium*? *gracilicaule*.- Gravier-Bonnet & Bourmaud, 2012: 106 [40].

*Gymnangium eximium*.- Ryland & Gibbons, 1991: 539–542: fig. 11 (in part, only form 2) [86].

*Halicornaria gracilicaulis gracilicaulis*.- Millard, 1968: 282–283 [91].

*Gymnangium gracilicaulis*.- Schmidt, 1972: 41, 43–44 [83]; Schmidt, 1973: 285 [104]; Mergner & Wedler, 1977: 24, pl. 6: fig. 39, pl. 9 fig. 61 [84].

*Gymnangium gracilicaule gracilicaule*.- Millard & Bouillon, 1973: 8, 91–92 [47]; Millard & Bouillon, 1974: 10 [46]; Millard, 1975: 443, fig. 136A, D [7]; Millard, 1978: 192 [105].

*Halicetta gracilicaulis*.- Hirohito, 1995: 293, figs.103b-d [20].

#### Material examined

Totally 52 colonies inspected (for sample coordinates, depths, collection numbers see S1 Appendix).

Molecular data available (see S1 Appendix for GenBank accession numbers.

Type locality: China Sea, 27°35'N; 123°35'E, 91m.

Distribution: tropical and subtropical waters of the western Pacific, Japanese waters (Sagami Bay) [103], the Indian Ocean, Maldives [40], Red Sea [84], Gulf of Suez [95,98], east coast of Africa, Natal, Mozambique [7], Seychelles [47], Zanzibar [48].

Depth: 42–350 m, present material: between 0–55 m.

#### Description

Colour in situ: skeleton bright, red-brown for stem, yellow-brown for branches, translucent for hydrocladia; coenosarc white, hydranth tentacles transparent.

Hydrorhiza root-like for attachment to hard substrata. Colony flexuous, size up to 30 cm (Figs 7b and 9a). Stem polysiphonic composed of several parallel tubes with primary axial tube running on the frontal surface. Short hydrocladia arising alternately from the axial tube, usually with 3 hydrothecal segments (sometimes up to 5).

Branches may be monosiphonic or polysiphonic in large colonies, reaching lengths of 18 cm. Monosiphonic branchlets up to 3.5 cm long, springing alternately from the accessory tubules from a long, sharply pointed segment forming a very oblique hinge-joint long, with up to 5 two-chambered nematothecae) (Fig 9c). Branchlets divided into internodes by oblique nodes, bearing hydrocladia usually curved and not in the plane of the colony but perpendicular to it.

Hydrocladia up to 1.7 mm long, with up to 7 hydrothecate internodes. Hydrothecate internodes with two perisarcal slight thickenings, one near the adcauline latero-basal thickening of the hydrothecal wall, a second at the level of the base of the lateral nematothecae.

Hydrotheca axis S-shaped, largest part of abcauline wall straight and parallel to internode; upper part bends to abcauline side, margin smooth, depth variable, adcauline lateral thickening of differing length at the level of lower internodal thickening (Fig 9b).

Median nematotheca perpendicular to internode or curved upwards, adnate to about half of its length, with two apertures, one terminal circular and one at the beginning of free part on upper surface, connection between nematotheca and hydrothecal cavity at the top of adnate part and between nematotheca and internode.

Lateral nematothecae tubular narrowing to the top, overtopping the level of hydrothecal aperture, facing upwards and forwards, with one external orifice on top, more often of larger size on the last hydrocladial segment.

Gonothecae borne on stem and hydrocladia, flattened and rounded, with a short peduncle and without distal horn-like processes (Fig 9d). Female with truncated distal end. A single oocyte per gonotheca.

Nematocysts: long and thin microbasic mastigophores gathered in bundles inside the lateral and median nematothecae.

Measurements given in S4 Appendix.

#### Remarks

Distinguishing *T*. *gracilicaulis* and *T*. *eximia* can be difficult and also the molecular data (Figs 4 and 5) do not resolve the two morphotypes as separate lineages, they both cluster in a single clade but without any apparent order. It is tempting to interpret this as evidence that both morphotypes belong to the same phylogenetic species. However, this could also be just a lack of resolution of the used 16S and CAM markers. A separate, more detailed examination including also more variable nuclear markers (i.e. microsatellite markers) is needed. For this, both morphotypes must unambiguously identifiable. Based on the material examined for this study, *T*. *gracilicaulis* can be distinguished from *T*. *eximia* based on the following differences: monosiphonic branchlets attached to an apophysis-like process ending with a hinge-joint, the more gracile, flexuous and delicate appearance of the colony, the perpendicular position of the hydrocladia and the more elongated hydrothecae (comp. Figs 8b and 9b). The apophysis-like process ending with a hinge-joint (Fig 9c) described by Jäderholm [92] was not always reported in subsequent descriptions of *T*. *gracilicaulis*.

Rees & Vervoort [48] and some other authors (see under *T*. *eximia*) observed lateral lobes on the hydrothecal margin of *T*. *eximia*, the feature that could be used to separate the species, but in the present material we did not note the presence of distinct lobes in *T*. *eximia*, as also noticed in Maldives specimens [40]. Another character given by Rees & Vervoort [48] to distinguish the two species, i.e., polysiphonic side-branches in *T*. *eximia* instead of strictly monosiphonic ones in *T*. *gracilicaulis* is also not applicable for the present material as the large *T*. *gracilicaulis* had polysiphonic stems and branches.

The nematothecae of the axial tube may have one or two external openings, this in difference to the description of Rees & Vervoort [48] who reported two openings for *T*. *gracilicaulis*.

*Taxella gracilicaulis* described by Billard [45] is probably in part *T*. *gracilicaulis* and in part *T*. *eximia*. The specimen from Macalonga ([45] figured on Plate 25, fig. 7) with its large, stiff colony resembling a Christmas tree branch and with its thick, polisiphonic branches is more likely *T*. *eximia*, while the other with looser branching fit to the description given by Jäderholm for *T*. *gracilicaulis*.

### *Taxella hornelli* (Thornely, 1904) new comb.

Fig 10a–10d

**Fig 10:**
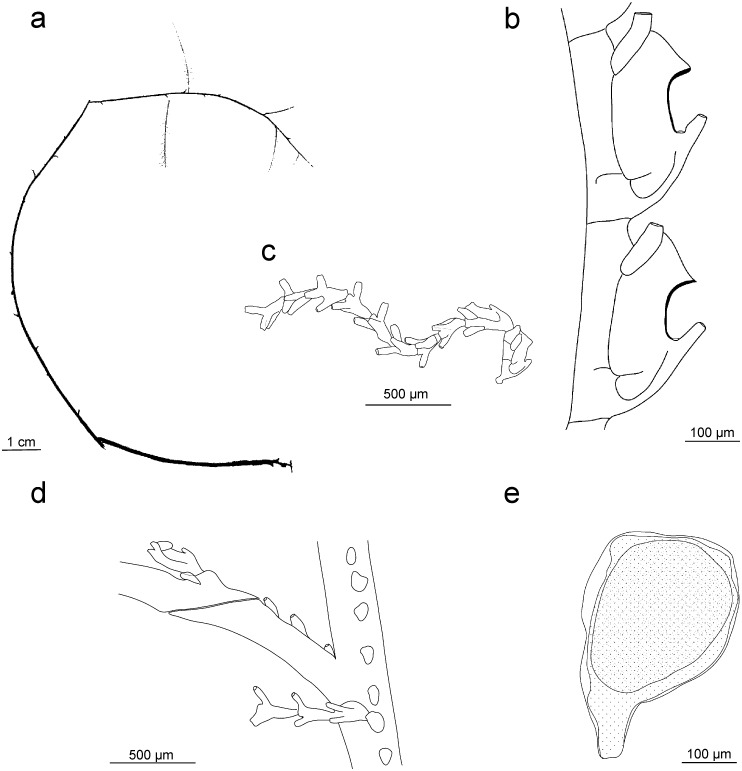
*Taxella hornelli*: a) colony, b) part of hydrocladium, c) pseudophylactocarp, d) oblique hinge joint in side-branch and front of stem, e) gonotheca.

*Lytocarpus* (?) *hornelli* Thornely, 1904: 123, pl. 3: figs 1-1b [78].

*Gymnangium hornelli*.- Gravier-Bonnet & Bourmaud, 2012: 106 and 113, fig. page 121 [40].

#### Material examined

Totally 4 colonies inspected (for sample coordinates, depths, collection numbers see S1 Appendix).

Molecular data available (see S1 Appendix for GenBank accession numbers.

Type locality: Ceylon.

Distribution: Ceylon [78], Maldives (present material).

Depth: 46–146 m, present material: 30–33 m.

#### Description

Colour in situ and preserved: skeleton bright yellow for the stem and branches, translucent to white for hydrocladia; coenosarc white, hydranth tentacles transparent.

Colony large, up to 29 cm, slender, flexuous, multipinnate, branched not in one plane (Fig 10a). Stem polysiphonic, smooth dorsally and provided with a primary tube that coils around the stem from the base to the distal end. Base of stem without any side-branches over a long distance (5 cm). Branches arranged spirally, mono- or polysiphonic, arising laterally from the secondary tubes. A large triangular and pointed apophysis-like segment at the base of branchlets bearing 2–3 nematothecae, sometimes segmented once or twice, ending by a deeply cut hinge-joint. Primary tube unsegmented, provided with nematothecae in a single row and with modified hydrocladia coiling around the fascicled stem. Two to four simple cauline nematothecae in between two successive modified hydrocladia plus one facing the point of their insertion.

Modified hydrocladia formed by a series of nematothecal segments or of 3 hydrothecal segments at base followed by a string of up to 16 nematothecal segments (Fig 10c), each bearing 3 tubular nematothecae inserted in a spiral and forming the longest part of the modified hydrocladia. Such pseudophylactocarps are often broken in preserved material and tend to fall during the collection as very fragile, even in living condition, but the proximal segment with hydrothecae usually remain attached to the stem.

Branches mono- or polysiphonic, but terminal branches monosiphonic, bearing hydrocladia not in one plane. A single nematotheca in between two successive hydrocladia. Apophysis of hydrocladia short, with one nematotheca at the base.

Hydrocladia curved, formed of up to 8 hydrothecate segments.

Hydrotheca with the adnate proximal part much longer than the free erected distal part (Fig 10b). Aperture circular, forming an angle of 35–45° with hydrocladial axis. Abcauline side below margin more or less thickened depending on location of the hydrotheca within colony (absent in most recently formed at end of branches). Rim even (no teeth), usually straight. A thin linear thickening of the skeleton present laterally on the sides of the walls at the base of the hydrotheca. A well-centred hydropore on the bottom, with abcauline desmocytes for the link of the hydranth present above.

Lateral nematothecae simple and tubular, tapering towards top and surpassing the hydrothecal rim, with two orifices, one on top and one basally facing the hydrocladium.

Median nematotheca short, tubular and tapering distally, provided with 3 apertures, the first connected with the inside of the hydrotheca, the second facing the hydrotheca on base, and the third at the top end of the tube.

Cauline nematothecae simple and more or less triangular, large on base and tapering on top in a tube facing laterally (Fig 10d).

Male gonothecae unprotected on short and straight peduncle, inserted in rows on branches, arising from hydrocladial apophysis, lentil-like, when fully-grown distal end truncated or rounded (Fig 10e). Female gonothecae were not observed. This is the first description of the gonosome of this species.

Measurements given in S4 Appendix.

#### Remarks

**Fig 11:**
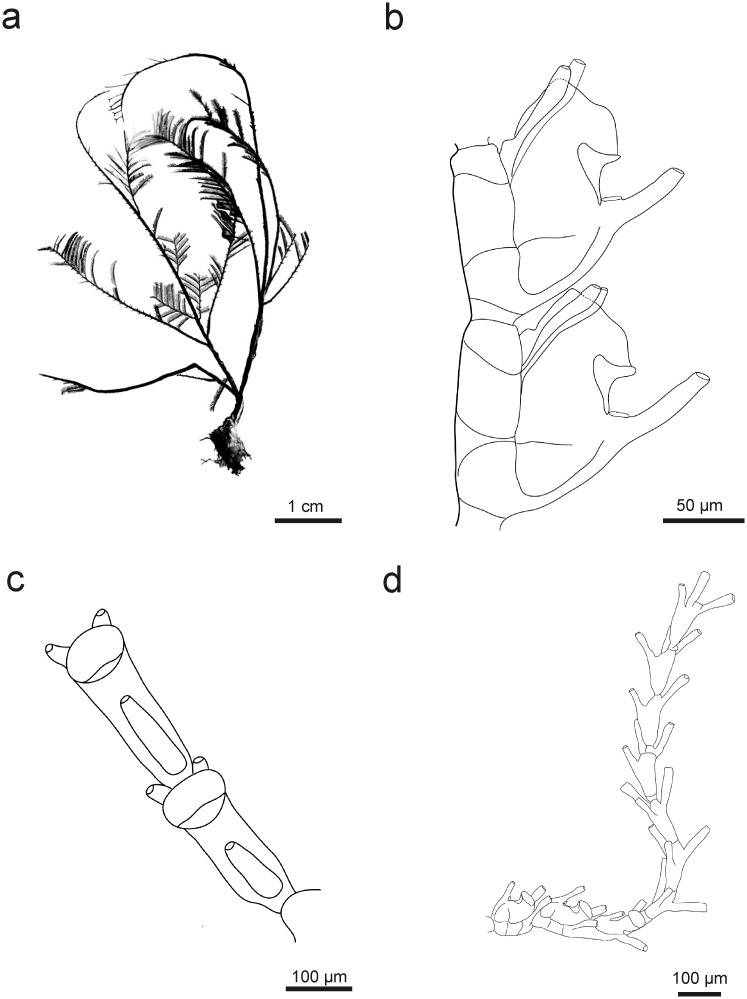
*Taxella longicornis*: a) colony silhouette, b) part of hydrocladium, c) part of hydrocladium—frontal view, d) pseudophylactocarp.

This species is very rare and has been recorded only a few times. The most characteristic feature is the presence of a linear appendages composed of nematothecate segments. These modified hydrocladia without gonothecae (observed also in *T*. *longicornis*) has been named pseudophylactocarp by Watson [58]. Thornely [78] described pseudophylactocarps with three segments, sometimes with up to twelve. Later, Thornely [101] found this species in the Red Sea but she declared that what she initially thought were strings of segments with nematothecae were actually complete hydrocladia, because “hydrothecae were also present along with nematophores”. This description is not clear, and adding to the confusion is the lack of a figure. It is possible that she could have observed *T*. *longicornis* instead of *T*. *hornelly* which have a pseudophylactocarp with several hydrothecate segments (Fig 11d).

Stechow [106] synonymised *Lytocarpus hornelli* with *L*. *(*?*) multiplicato-pinnata* (Kirchenpauer, 1876). As Kirchenpauer did not provide a description, Stechow based his opinion on a comparison with the type specimen (microscopic slide). Stechow, however, did not mention about pseudophylactocarps and the characteristic hinge-joint what makes this synonymy uncertain.

*Lytocarpus hornelli* described by Jarvis [81] does not agree with our current concept of *T*. *hornelli* for several diagnostic characters. Jarvis’ [81] specimen had a regularly branched colony in contrast to the loose to bottle-brush form of *T*. *hornelli*, hinge-joints and pseudophylactocarp are absent, and the median nematothecae are much longer and diverged at greater angle. More probably, this is a different species, but a reliable identification is not possible.

### *Taxella longicornis* (Busk, 1852) new comb.

Fig 11a–11d

*Plumularia longicornis* Busk, 1852 [107].

*Lytocarpus longicornis*.- Allman, 1883: 45, pl. 19: figs 4–6 [108].

*Aglaophenia longicornis*.- Bale, 1884: 157, pl. XIV: figs 7–8, pl. XVII: fig.5 [61].

*Halicornaria intermedia*.- Billard, 1913: 65, text-fig. 53, pl. 4: fig. 37 [45], Van Soest, 1976: 87 [109].

*Halicornaria longicornis var*. *sibogae* Billard, 1913: 67 [45].

*Macrorhynchia* (?) *longicornis*.- Stechow & Müller, 1923: 474 [110].

*Halicornaria longicornis*.- Briggs & Gardiner, 1931: 195, fig. 6 [111]; Pennycuik, 1959: 186 [112].

*Gymnangium longicorne*.- Watson, 2000: 60, fig. 48 [58]; Schuchert, 2003: 218, fig. 65 [113].

#### Material examined

Totally 3 colonies inspected (for sample coordinates, depths, collection numbers see S1 Appendix).

Molecular data not available.

Type locality: Torres Strait.

Distribution: Torres Strait (between Australia and New Guinea) [107], east coast of Australia [61], Darwin Harbour (north Australia) [58], Penguin Channel [111], Queensland [112], Kei Islands, Indonesia [113], Réunion (present study).

Depth: 3–50 m, present material: between 30–55 m.

#### Description

3 large, infertile, irregularly ramified colonies up to third degree, multipinnate (16.5 cm, 15 cm high) (Fig 11a).

Stem polysiphonic, composed of primary tube and several accessory tubes. Primary tube of stem with alternate, short hydrocladia of up to 3 hydrothecae. Primary branches polysiphonic, arising from the accessory tubes, not segmented, up to 12 cm long. Branchlets (last order branches) up to 1.5 cm, monosiphonic, with short, pointed first segment bearing up to 2 nematothecae, ending in oblique hinge-joint, following segments with one hydrocladium per internode. Hydrocladia arranged alternately, up to 7 hydrothecate segments, not in one plane but directed upward at an angle of 90° and less.

First or second hydrocladium on branchlet (sometimes both) modified into long pseudophylactocarp consisting of 3 to 4 hydrothecate segments and a row of up to 18 segments with 3 nematothecae each (Fig 11d).

Apophysis of hydrocladia associated with two nematotheca, one at the base and one axillary, both with two openings.

Hydrotheca deep, distal part sharply bent at an angle of about 45° to almost 90° to hydrocladial axis, with abcauline wall strongly thickened at the level of bent, rim with one broad lateral lobe, adcauline lateral thickening of variable length near base (Fig 11b and 11c).

Lateral nematothecae long and tubular, extending beyond hydrothecal rim, with two orifices, one on top and one basally facing the hydrocladium.

Median nematotheca of variable length, usually very long, tubular, with 3 apertures, the first connected with the inside of the hydrotheca, the second on upper side of free part, and the third at the top end of the tube.

Gonothecae not present in the material (for description see Schuchert [113]).

Measurements given in S4 Appendix.

#### Remarks

*Taxella longicornis* has recently been re-described by Watson [58] and Schuchert [113].

Although the species was not included in the molecular analysis, it was placed in the genus *Taxella* because it matched our new, emended diagnosis.

### *Taxella elfica* Ronowicz sp. nov.

urn:lsid:zoobank.org:act:D5BCAF6E-393A-435E-9E63-426FD3B415B6

Fig 12a–12f

**Fig 12:**
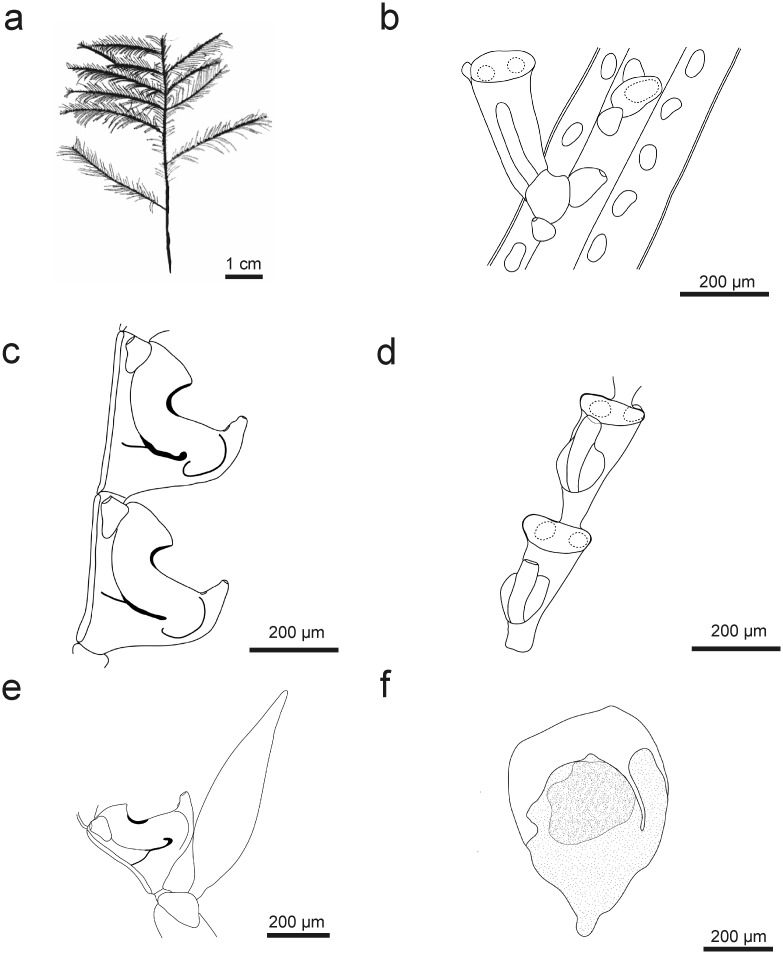
*Taxella elfica* sp. nov.: a) colony silhouette, b) part of stem, two apophyses with basal and axillar nematothecae and one hydrotheca and rows of nematothecae on axillary tubes, c) hydrothecae—lateral view, d) hydrothecae—frontal view, e) apophysis with gonotheca (lateral side) and hydrotheca, f) female gonothecae.

#### Material examined

Type material

Holotype colony MHNG-INVE-36231; Madagascar; 17.06.1965; 60m; 2 stems: 7 cm long and a larger one broken, with gonothecae.

Paratypes MHNG-INVE-36266; Madagascar, Mitsio; 12°86'S, 48°59'E; 28.07.1958; 64m; 3 large infertile colonies.

Totally 5 colonies inspected.

Molecular data not available.

Type locality: Madagascar.

Distribution: known only from the type locality.

Depth: 60 m and 64 m.

#### Etymology

The name is an allusion to the shape of an elf shoe, to which the form of the hydrotheca resembles.

#### Description

Colony large, 7 cm long with root-like hydrorhiza. Stem branched regularly with opposite side branches, multipinnate (Fig 12a). Stem and side-branches polysiphonic, up to 800 μm thick, with primary axial tube unsegmented, bearing alternate apophyses for the attachment of hydrocladia and a row of nematothecae, one basal and one axillary to each apophysis (Fig 12b).

Side-branches opposite, polysiphonic up to 40 mm long, arising from the accessory tubes without apophyses. Accessory tubes with rows of nematothecae (Fig 12b). Primary tube of branches not segmented, bearing densely set alternate hydrocladia, with four or five nematothecae below first hydrocladium.

Hydrocladia 3 mm in length, segmented, separated by oblique nodes, and with up to 18 hydrothecate segments. Strong thickening at the lower third part of each internode not always present.

Hydrothecae large, deep and wide, upper part parallel to hydrocladial axis, basal 2/3 curved away from axis, almost horizontal and bulging protruding from the hydrocladium (Fig 12c). Aperture making an angle of ca. 45° with the hydrocladial axis, below aperture abcauline side curved and thickened but not forming a septum. Hydrothecal margin smooth (Fig 12d).

Median nematothecae pointing obliquely forwards, with two external openings: one at distal end and one on upper side of free part, one internal connection with inside of hydrotheca and one—with internode.

Lateral nematothecae not reaching beyond margin of hydrotheca, simple, tubular, with one opening directed rearward and one internal connection with hydrotheca.

Gonothecae borne on apophyses of stem and branches, rounded and flattened, with short pedicel, top truncated (Fig 12e and 12f).

Measurements given in S4 Appendix.

#### Remarks

In Madagascar another similar *Taxella* species may be found—*T*. *gracilicaulis*. *Taxella* sp. nov. is easily distinguishable by thick polysiphonic stem, opposite polysiphonic branching, and the bulging hydrothecae with their base erected from the hydrocladium.

### Genus *Gymnangium* Hincks, 1874 emended

**Diagnosis:** Aglaopheniidae with unprotected gonothecae, colonies monosiphonic or exceptionally polysiphonic, with cup-shaped hydrothecae. Type species *Halicornaria montagui* Billard, 1912 by designation of Stechow (1923: 236) [11].

### *Gymnangium hians* (Busk, 1852)

**Fig 13:**
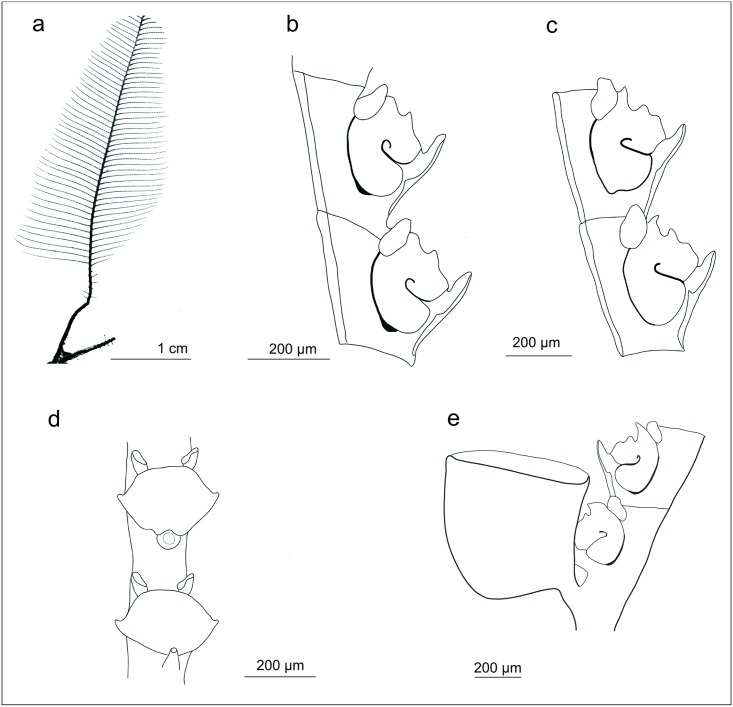
*Gymnangium hians*: a) colony silhouette, b) hydrothecae with two marginal teeth,–lateral view, c) hydrothecae with three marginal teeth, d) hydrothecae—frontal view, e) gonothecae.

Figs 7c, 7d and 13a–13e

*Plumularia hians* Busk, 1852: 396 [107]; Kirchenpauer, 1876: 30 [73].

*Halicornaria hians*.- Bale, 1884: 179–180, pl. 13: fig. 6, pl. 16: fig. 7 [61]; Kirkpatrick, 1890b: 604 [114]; Stechow, 1909:101, pl. 1: fig. 11, pl. 6: figs. 16,17 [115]; Billard, 1913: 68,69 [45]; Stechow, 1913: 94–95, fig. 61 [116]; Jäderholm, 1916: 8, fig. 5 [117]; Briggs, 1918: 47 [118]; Stechow, 1919: 125 [106]; Bedot, 1921: 347 [76]; Jarvis, 1922: 355 [81]; Nutting, 1927: 237 [67]; Vervoort, 1941: 222–225, figs. 7–8 [119]; Millard, 1958: 219–220, fig. 15G-H [80]; Pennycuik, 1959: 186 [112].

*Aglaophenia balei* Marktanner-Turneretscher, 1890: 272–273, pl. 7: figs. 19–20 [79]; Billard, 1905: 334 [120].

*Halicornaria flava* Nutting, 1906: 955, pl. 6: fig. 2, pl. 13: figs. 11–12 [121].

**Fig 14:**
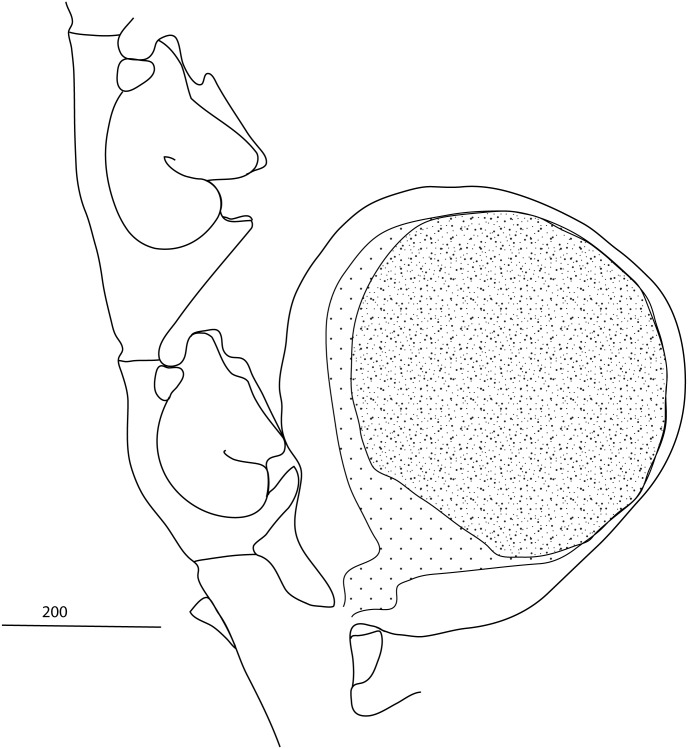
*Gymnangium bryani*: Part of stem with gonothecae.

*Halicornaria hians* var. *profunda* Ritchie, 1909: 528 [122]; Ritchie, 1910: 24, pl. 4: figs. 13–14 [94]; Jäderholm, 1919: 26, pl. 6: fig. 6 [96].

*Halicornaria balei*.- Ritchie, 1910: 22–23 [94].

*Halicornaria balei* var. *flava*, Nutting (?) Ritchie, 1910: 23, pl. 4: fig. 12 [94].

*Halicornaria haswellii* Bale, 1884: 180–181, pl. 13: fig. 5, pl. 16: fig. 8 [61].

*Halicornaria hians* var. *laxa* Ritchie, 1910: 835–836, fig. 81 [123]; Rees & Thursfield, 1965: 197 [102]; Smaldon, Heppell & Watt, 1976: 23 [124].

*Halicornaria hians* var. *balei* Billard, 1913: 70, fig. 56 [45]; Bedot, 1921: 347 [76]; Van Gemerden- Hoogeveen, 1965: 70–73, figs. 39–41 [125].

*Halicornaria hians* var. *flava*.- Bedot, 1921: 347 [76].

*Gymnangium hians*.- Stechow, 1923: 19 [126]; Stechow, 1923: 236, 239 [11]; Stechow, 1924: 69 [127]; Stechow, 1925: 254 [128]; Yamada, 1958: 51, 61–62 [129]; Yamada, 1959: 84 [19]; Ooishi, 1964: 191 [130]; Rees & Thursfield, 1965: 171 [102]; Rho, 1967: 346, fig. 6 [131]; Millard & Bouillon, 1973: 92–93 [47]; Rho & Chang, 1974: 147 [132]; Millard, 1975: 444–445, fig. 134G-H [7]; Rho, 1977: 279, 425, pl. 93: fig. 93 [133]; Vervoort & Vasseur, 1977: 84–86, fig. 35 [85]; Millard, 1978: 193 [105]; Hirohito, 1983: 77 [103]; Rees & Vervoort, 1987: 172–175, fig. 41 [48]; Hirohito, 1995: 287–290, fig. 101 a.b [20]; Watson, 2000: 58–60, fig. 47 [58]; Vervoort & Watson, 2003: 292, fig. 69F [57].

*Gymnangium hians* var. *balei*.- Schmidt, 1972a: 41 [83]; Mergner & Wedler, 1977: 24, pl. 6: fig. 38, pl. 10: figs. 64–69 [84].

*Gymnangium hians* var. *flava*.- Mammen, 1967: 311 [82].

#### Material examined

Totally 126 colonies inspected (for sample coordinates, depths, collection numbers see S1 Appendix).

Molecular data available (see S1 Appendix for GenBank accession numbers.

Type locality: Torres Strait (16 m)–between Australia and Papua New Guinea.

Distribution: Indo-Pacific: South Africa (Natal) [7]; East Indies, Japan, Hawaii, Indian Ocean, Red Sea, Sagami Bay [115], China Sea [67].

Depth: 0–73 m, present material: between 0–60 m.

#### Description

Colour in situ: stem from light brown to blackish, hydrocladia lighter, taking the colour of the coenosarc from cream-coloured to dark green.

Colonies stiff in the form of monosiphonic, unbranched, pinnate stems (Figs 7c, 7d and 13a). The maximum colony height 19 cm. Stem internodes not visible. Hydrocladia up to 2.5 cm long in large colonies, alternate, pointing to the left and right from the axis of stem. At the base of each hydrocladium three nematothecae, two on the anterior side: axillary and basal and one axillary, on the posterior side. Hydrocladia segmented by slightly oblique nodes, no internodal thickenings.

Hydrothecae cup-shaped, inclined about 45° to the axis of the stem, lateral margin with two or three pairs of distinct lateral cusps, when two cusps present, then located in the adcauline half of the thecal margin, an abcauline intrathecal septum reaching about half-way across, thickened and curved upwards at the end where skeleton protuberances occurs, spines around the hydropore at the bottom of hydrothecae sometimes present (Fig 13b and 13c). Median nematothecae of variable length, pointed at the distal part, without any internal connection with hydrothecae, adnate to the level of hydrothecal margin, extending beyond hydrothecal rim in a gutter-shaped opening until top.

Lateral nematothecae sac-shaped, reaching hydrothecal margin, variable in length—sometimes overtopping the level of longest tooth, sometimes not, with one distal opening (Fig 13d).

Gonothecae forming a double row on front of stem (Fig 7d), settled on a short and lateral peduncle arising from hydrocladial apophysis, conical with circular section, truncated distally (Fig 13e). Gonophores with free medusoids.

Measurements given in S4 Appendix.

#### Remarks

This is morphologically a very variable species. Ritchie [94,122,123] introduced *Halicornaria hians* varieties “*profunda*” and “*laxa*” based on differences in length of hydrothecal depth and length of hydrothecal articles. Billard [45] and later Vervoort [119] pointed out that intra-population variability of these characters argues against a formal recognition of these subspecies. Pennycuik [112] synonymised *H*. *haswelli* Bale, 1884 with *H*. *hians* because the characters given by Bale [61] were found to be variable even within one colony: (i) the closeness of hydrothecae, (ii) the size of teeth, (iii) the presence of two apertures in median nematothecae.

### *Gymnangium bryani* (Nutting, 1906)

Fig 14

*Halicornaria bryani* Nutting, 1906: 956, pl. 4: fig. 4, pl. 13: figs 13–14 [121].

*Gymnangium hians*.- (in part) Millard & Bouillon, 1973: 93 [47].

#### Material examined

Totally 5 colonies inspected (for sample coordinates, depths, collection numbers see S1 Appendix).

Molecular data not available.

Type locality: Kauai (Hawaii) [121].

Distribution: Hawaii, Mozambique Channel (Glorieouses, Mayotte), common in North Sulawesi, epizootic on *Aglaophenia cupressina* (Di Camillo, personal communication).

Depth: 390 m, present material: between 5–15 m.

#### Description

Colony small, epizootic on *Aglaophenia cupressina* or *Macrorhynchia spectabilis* (Allman, 1883), with creeping Hydrorhiza attached to the host species, erect hydrocladia arising directly from stolon without apophysis, up to 1.5 mm high, segmented through slightly oblique internodes (Fig 14).

First segment of hydrocladium sometimes longer and lacking hydrothecae, with one or two nematothecae.

Hydrothecae margin making an angle of ca. 45° to almost 90° with the hydrocladial axis, abcauline side curved and thickened below aperture, forming an intrathecal septum reaching half way across the hydrotheca and curved upwards at the end, rim variable from no tooth to 2 or 3 teeth.

Median nematotheca triangular and pointed, gutter shape, free part very short.

Lateral nematothecae very short, triangular, not reaching hydrothecal margin.

Gonothecae arising from the first or second athecate segment by the mean of a short lateral peduncle, large, conical to barrel-shaped, truncated distally with a rim prominent like in the Nutting’s figure [121]. On the same segment there are two nematothecae: one below pedicel on the anterior side and another one above pedicel, on the posterior side.

Gonophores with medusoid inside (release not observed).

Measurements given in S4 Appendix.

#### Remarks

This is an epizootic species found on *Aglaophenia cupressina* and *Macrorhynchia spectabilis*. It is distinct from *G*. *hians* because the colony is not feather-like, it gets reproductive at a very small size, and the gonothecae arise from the first segments of the hydrocladia. Unfortunately no alcohol sample was available to provide molecular data to study the relationship to *G*. *hians*, which has similar hydrothecae. It is possible that this is just an epizootic variety of *G*. *hians*.

### *Gymnangium millardi* Ronowicz sp. nov.

urn:lsid:zoobank.org:act:460E0A40-46BD-464E-B3FC-AABD286E74BF

Fig 15a–15d

**Fig 15:**
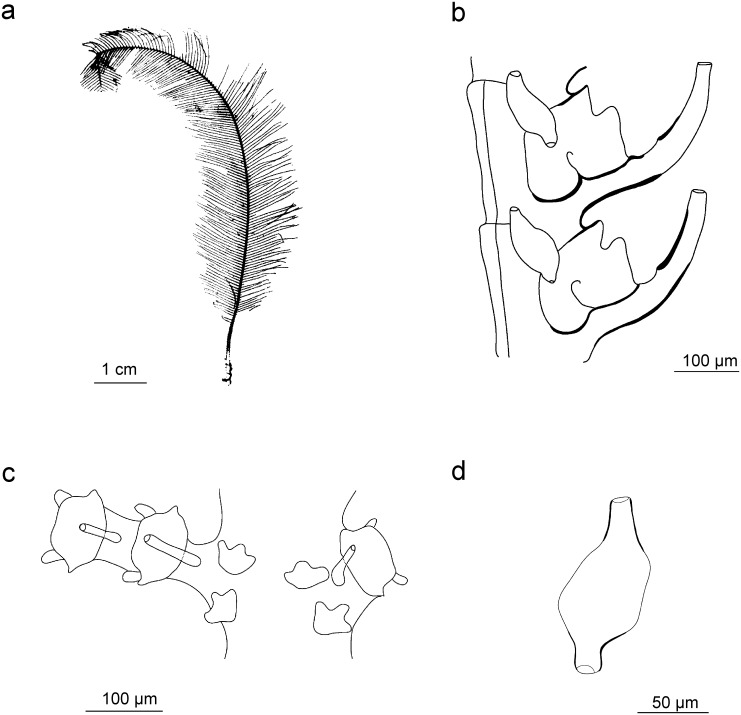
*Gymnangium millardi* sp. nov.: a) colony silhouette, b) hydrothecae—lateral view, c) part of stem with hydrothecae (frontal view) and cauline nematothecae, d) lateral nematotheca.

*Halicornaria allmani* Millard, 1968: 282, fig. 6C [91].

*Gymnagium allmani* Millard, 1975: 434, fig. 134J—K [7].

[Not *Gymnangium allmani* (Marktanner-Turneretscher, 1890) [79]]

#### Material examined

Type material

Holotype colony SAM-MB-H001923; North of Durban; 28°13'S, 32°34'E; 27.01.1975; 48m; fertile, alcohol sample and slide.

Paratypes SAM-MB-H003480; 25°57'S, 33°20'E; 08.08.1964; 42m; infertile; alcohol sample and slide.

Eur11_180; NGB&CB collection; Europa; 22°38'32"S, 40°38'51"E; 10.11.2011; 13m; infertile.

Mad71_34; NGB&CB collection; Madagascar (Tulear); 1971; 34m; infertile.

Totally 2 colonies inspected.

Molecular data available (see S1 Appendix for GenBank accession numbers.

Type locality: South Africa.

Distribution: Natal, South Africa and Mozambique [7,91]; Europa (Mozambique Channel) and Madagascar (present study).

Depth: 42–64 m, present material: 13–34 m.

#### Etymology

The species name *millardi* has been chosen in honour of Dr. Naomi Millard and her outstanding work on hydroids from southern Africa and some other Indian Ocean locations.

#### Description

Monosiphonic, pinnate stem, 3 cm high, unbranched, segmented, divided by oblique nodes, bearing alternate hydrocladia, one or two on internode directed upwards at an angle of ca. 60° to axis (Fig 15a).

Hydrocladia divided into internodes by distinct, straight to slightly oblique nodes (Fig 15b). Each hydrocladial apophyses with three nematothecae, one axillary, on the posterior side of stem with 3 to 4 apertures, two on the anterior side, axillary one with three to four apertures and basal one with three apertures (Fig 15c).

Hydrothecae cup-shaped, free adcauline wall short and thickened, abcauline wall completely adnate to median nematotheca, thickened, margin with one distinct lateral cusp and deep notch located at the adcauline half of thecal margin, together with sharply curved adcauline hydrothecal rim it looks as if there are two teeth on each side, an abcauline intrathecal septum thickened and curved at the end, reaching about half-way across the lumen of the hydrotheca (Fig 15b).

Median nematothecae tubular, very long, projecting beyond hydrothecal margin for about the same length as the hydrothecal depth, with two orifices, one terminal and one just above junction with hydrotheca, no communication with hydrothecae.

Lateral nematothecae very characteristic, tubular, not reaching to thecal margin, with two apertures, one facing upward, one opposite facing downward (Fig 15b and 15d), sometimes a third aperture occurs in the middle of the nematotheca. Gonothecae forming two rows on front of stem, sac-shaped and truncated distally [7], absent in the present material.

Measurements given in S4 Appendix.

#### Remarks

The present material largely agrees with *G*. *allmani* described and illustrated by Millard [7,91]. Millard [91] admitted that her specimen combined features of both *G*. *allmani* (Marktanner-Turneretscher, 1890) and *G*. *montagui* (Billard, 1912). The hydrothecal margin resembled *G*. *montagui* in the shape of lateral tooth, which was straight or oblique but not recessed and the bay behind was shallow while other features like cauline nematothecae with more than one aperture and tubular median nematothecae was like in *G*. *allmani*.

Galea [6] provided a detailed re-description of *G*. *allmani* and suggested that the African hydroids described by Millard do not belong to *G*. *allmani* due to some differences such as: 1) the presence of one hydrocladium per stem internode, 2) the hydrothecal margin with one low but distinct tooth and a bay of varying depth posterior to it, 3) the structure of lateral nematothecae which are situated on a tubular neck. Millard [7] noted a variation in the two first characters. Our scarce material unfortunately does not allow us to study the variation between specimens. However comparing existing descriptions of *G*. *allmani* in the literature point a unique trait which is also found in the present material and Millard’s specimens: lateral nematothecae with two openings at ends of two opposite tubular necks, sometimes a third opening in the middle of nematotheca (Fig 15d).

Although the species was recorded and described previously by Millard [7], but the given name was not accurate, we suggest to use a new name *G*. *millardi* in honour of the author of its first description.

### *Gymnangium ferlusi* (Billard, 1901)

Fig 16a–16f

**Fig 16:**
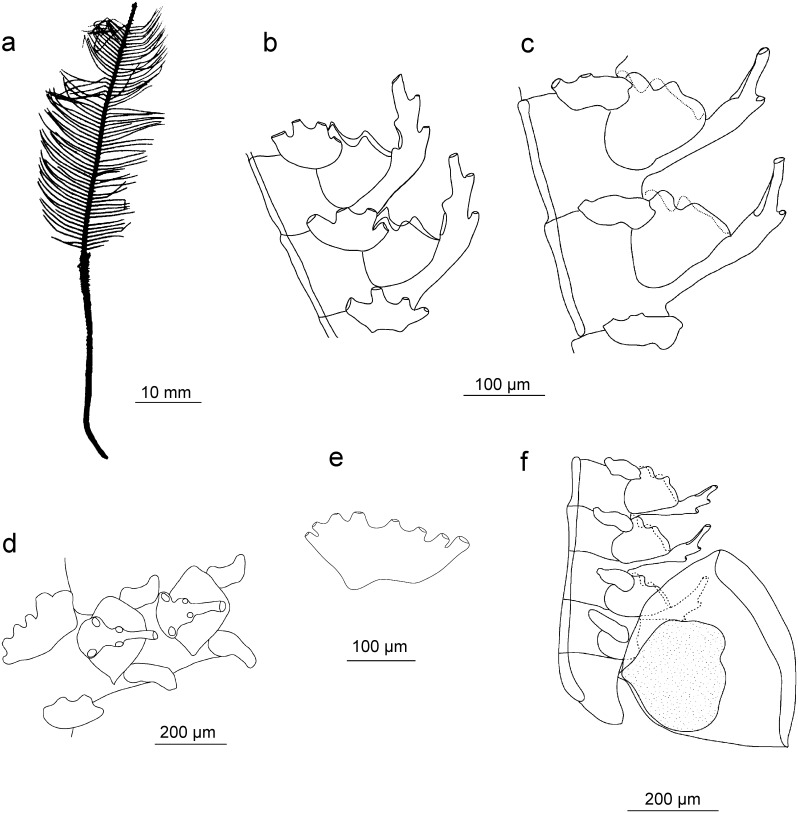
*Gymnangium ferlusi*: a) colony silhouette, b-c) hydrothecae—lateral view, d) hydrothecae—frontal view and two cauline nematothecae, e) posterior axillary cauline nematotheca, f) part of hydrocladium with gonotheca.

*Halicornaria ferlusi* Billard, 1901: 121, figs 3–4 [134]; Billard, 1907: 370, fig. 14, pl. 25, fig. 8 [45]; Millard, 1962: 312 [135]; Gravier-Bonnet, 1979: 62–67, figs. 12–13 [136]; Van Praët, 1979: 912, fig. 76 [137].

*Gymnangium (Haliaria) ferlusi*.- Stechow, 1921: 234 [15].

*Halicornaria ferlusi* var. *brevis* Jarvis, 1922: 354, fig. 5, pl. 26, fig. 27 [81].

*Gymnangium ferlusi*.- Stechow, 1923: 237 [11]; Millard, 1975: 440, figs 137A-C [7]; Millard, 1978: 192 [105]; Rees & Vervoort, 1987: 166–168, fig. 39 [48].

#### Material examined

Totally 9 colonies inspected (for sample coordinates, depths, collection numbers see S1 Appendix).

Molecular data not available.

Type locality: Fort Dauphin, Madagascar [134].

Distribution: Madagascar [134], Wasin (east Africa) [81], South Africa [7,105], Arabian Sea [48].

Depth: 27–115 m, present material: 34–60 m.

#### Description

Colony pinnate, monosiphonic, unbranched, up to 12 cm long and 2 cm wide (Fig 16a). Stem segmented, bearing alternate hydrocladia. Hydrocladia with up to 58 thecate internodes divided by slightly oblique nodes (Fig 16b and 16c).

Each stem segment delimited by oblique nodes, with one apophysis for hydrocladial attachment and three cauline nematothecae, two on the anterior side, axillary one with three to four apertures and basal one with three apertures (Fig 16d) one axillary on the posterior side of stem with 3 to 8 apertures (Fig 16e).

Hydrothecae cup-shaped, widening to margin, without an abcauline intrathecal septum, margin with 2 pairs of lateral teeth and one small pointed abcauline tooth making an angle of 50° with axis of internode (Fig 16b and 16c).

Median nematotheca adnate to about half of its length with free part curved upwards and extending beyond hydrothecal border for about the same length as adnate part, tubular and branched, the main tube giving rise to 2 or 4 shorter lateral tubes located on abcauline side, with one large opening on upper surface at the beginning of the free part and 3 or 5 terminal apertures at the end of the tubes depending of their number, no connection with hydrotheca. Lateral nematothecae not reaching thecal margin, wide with four or five apertures on upper surface, of similar shape as cauline nematothecae.

Gonothecae forming a double row on frontal side of stem, arising from the hydrocladial apophysis, conical and truncated distally (Fig 16f).

Measurements given in S4 Appendix.

#### Remarks

The characteristic features of this species are very long and branched median nematothecae with its distinctive openings on the abcauline side as well as the oblong lateral and cauline nematothecae with many openings. The number of openings of the lateral- and cauline nematothecae varies between specimens. Likewise, also the number of openings of the median nematotheca varies between different colonies.

### *Gymnangium expansum* (Jäderholm, 1903)

**Fig 17:**
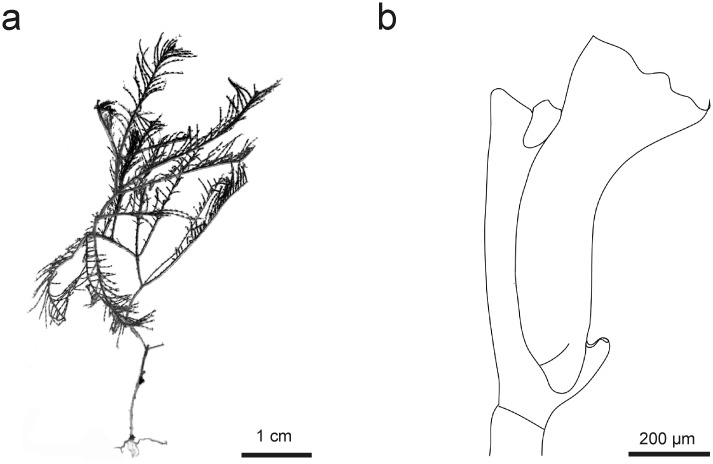
*Gymnangium expansum*: a) colony silhouette, b) hydrotheca—lateral view.

Figs 1b, 17a and 17b

*Halicornaria expansa* Jäderholm, 1903: 303–304, pl. 14 figs. 5–7 [71]; Stechow, 1907: 200 [65]; Stechow, 1909: 5, 103–104, fig. 8 [115]; Stechow, 1913: 10 [116]; Jäderholm, 1919: 26, pl. 26 fig. 7 [96]; Bedot, 1921: 346 [76]; Von Schenck, 1965: 942 [77].

*Halicornaria sibogae* Billard, 1918: 25–26, fig. 4 [138]; Rees & Vervoort, 1987: 165 [48].

*Halicetta expansa*.—Stechow, 1923: 19 [126]; Yamada, 1959: 83 [19]; Hirohito, 1995: 293, fig. 103 [20].

*Gymnangium expansum*.—Vervoort, 1966: 165, figs 65–66 [56]; Rees & Vervoort, 1987: 163–166, fig. 38 a,b [48]; Vervoort & Watson, 2003: 288–290, figs 68g, 69a [57]; Schuchert, 2015: 357, fig. 29 [139].

#### Material examined

Totally 1 colony inspected (for sample coordinates, depths, collection numbers see S1 Appendix).

Molecular data available (see S1 Appendix for GenBank accession numbers).

Type locality: Southern Japan

Distribution: Japan [20,71,139], New Zealand [57], east Africa [48].

Depth: shallow waters—741 m, present material: 141–165 m.

#### Description

For full description see Rees & Vervoort [48].

#### Remarks

A characteristic species with monosiphonic, branching, helicoidally arranged stem and very long hydrothecae without an abcauline septum (Fig 17a and 17b).

This is a type species of the genus *Halicetta* proposed by Stechow [15]. The morphological characters agree with those given by Stechow, i.e. “hydrothecae elongated, slightly curving but not bent, and without an abcauline intrathecal septum”. Stechow [15] included also species in *Halicetta* which are here placed in a separate genus *Taxella*. The 16S and Calmoduoin sequence data clearly placed *G*. *expansum* in the *Gymnangium* clade and not in the *Taxella* clade (Figs 4 and 5). Additionally, there is a different colony arrangements; the stem is monosiphonic, thus different from all *Taxella* species.

## Supporting information

S1 Appendix List of samples with museum accession numbers, GenBank numbers, and geographical coordinates for each station. (DOC)

S2 Appendix Parameters used in the PCA analysis. PC1, PC2 and PC3 indicate the respective weights of each parameter in the PCA.
(DOCX)

S3 Appendix Listing of 25 characters employed in the Principal Components Analysis (PCA). (XLS)

S4 Appendix Measurements of species of genera *Taxella* and *Gymnangium*. (DOC)
