# Supplementary material for: Modern alongside traditional taxonomy—Integrative systematics of the genera Gymnangium Hincks, 1874 and Taxella Allman, 1874 (Hydrozoa, Aglaopheniidae)
Source: PLoS One. 2017 Apr 19;12(4):e0174244. doi: 10.1371/journal.pone.0174244 (PMC5396908; doi:10.1371/journal.pone.0174244)
Supplement: S1 Appendix — (DOC) [file pone.0174244.s001.doc]

**S1 Appendix. List of samples with museum accession numbers, GenBank numbers, and geographical coordinates for each station.** Abbreviations: Mad – Madagascar, May- Mayotte, Mal – Maldives, Eur – Europa, Jdn – Juan de Nova, Glo – Glorieuses, Reu – Reunion, Gey – Geyser; MHNG – Natural History Museum of Geneva, MNHN – National Museum of Natural History, Paris, SAM – South African Museum, NGB&CB – Nicole Gravier-Bonnet and Chloé Bourmaud collection.

| ***Species*** | **Museum collection** | **GenBank**  **16S** | **GenBank**  **CAM** | **Sample** | **Locality** | **Latitude** | **Longitude** | **Date** | **Depth [m]** | **Fertility** |  |
| --- | --- | --- | --- | --- | --- | --- | --- | --- | --- | --- | --- |
| *Taxella eximia* | NGB&CB collection | KU594397 | KM587282 | Reu11_374 | Réunion | 21°16'43" S | 55°48'33" E | 2011.11.29 | 20-30 | yes |  |
| *Taxella eximia* | NGB&CB collection | KU594396 | KY117112 | Reu11_541 | Réunion | 21°16'43" S | 55°48'33" E | 2011.11.02 | 20-30 | yes |  |
| *Taxella eximia* | NGB&CB collection | KU594374 | KM587285 | Gey08_009 | Geyser | 12°20'48" S | 46°26'25" E | 2008.04.21 | 8-10 |  |  |
| *Taxella eximia* | NGB&CB collection | KU594376 | KM587286 | Gey08_013 | Geyser | 12°20'48" S | 46°26'25" E | 2008.04.21 | 8-10 |  |  |
| *Taxella eximia* | NGB&CB collection | KU594369 | KM587288 | Gey08_022 | Geyser | 12°20'48" S | 46°26'25" E | 2008.04.21 | 8-10 |  |  |
| *Taxella eximia* | NGB&CB collection | KU594375 | KM587287 | Gey08_095 | Geyser | 12°19'49" S | 46°27'63" E | 2008.01.22 | 8 |  |  |
| *Taxella eximia* | NGB&CB collection | KU594399 | KY117113 | May07_018 | Mayotte | 12°52'33" S | 45°16'52'' E | 2007.11.29 | 0-36 | no |  |
| *Taxella eximia* | NGB&CB collection | KU594410 |  | Eur09_056 | Europa | 22°20'29" S | 40°20'08" E | 2009.04.23 | 12-20 | no |  |
| *Taxella eximia* | NGB&CB collection | KU594405 | KM587291 | Eur09_057 | Europa | 22°20'29" S | 40°20'08" E | 2009.04.23 | 12-20 | no |  |
| *Taxella eximia* | NGB&CB collection | KU594410 | KM587277 | Eur09_058 | Europa | 22°20'29" S | 40°20'08" E | 2009.04.23 | 12-20 | no |  |
| *Taxella eximia* | NGB&CB collection | KU594405 |  | Eur11_029 | Europa | 22°19'77" S | 40°21'90" E | 2011.11.07 | 9-12 |  |  |
| *Taxella eximia* | NGB&CB collection |  | KM587284 | Eur11_084 | Europa | 22°20'44" S | 40°20'23" E | 2011.11.08 | 10-13 | yes |  |
| *Taxella eximia* | NGB&CB collection | KU594404 | KM587276 | Eur11_240 | Europa | 22°21'54" S | 40°19'78" E | 2011.11.11 | 12-17 |  |  |
| *Taxella eximia* | NGB&CB collection | KU594388 | KY117114 | Jdn09_021 | Juan de Nova | 17°03'44" S | 42°47'04" E | 2009.04.26 | 12-20 | no |  |
| *Taxella eximia* | NGB&CB collection | KU594387 | KM587293 | Jdn09_062 | Juan de Nova | no data | no data | 2009.04.27 | 2-4 | yes |  |
| *Taxella eximia* | NGB&CB collection | KU594387 | KM587294 | Jdn09_063 | Juan de Nova | no data | no data | 2009.04.27 | 2-4 | yes |  |
| *Taxella eximia* | NGB&CB collection | KU594387 | KM587295 | Jdn09_064 | Juan de Nova | no data | no data | 2009.04.27 | 2-4 | yes |  |
| *Taxella eximia* | NGB&CB collection | KU594387 | KM587296 | Jdn09_065 | Juan de Nova | no data | no data | 2009.04.27 | 2-4 | yes |  |
| *Taxella eximia* | NGB&CB collection | KU594381 |  | Jdn09_127 | Juan de Nova | 17°04'29" S | 42°45'58" E | 2009.04.28 | 10-20 | yes |  |
| *Taxella eximia* | NGB&CB collection | KU594416 |  | Mal09_179 | Maldives | 5°17'03" S | 73°15'62" E | 2009.05.29 | 30 | yes |  |
| *Taxella eximia* | NGB&CB collection | KU594413 |  | Mal09_180 | Maldives | 5°17'03" S | 73°15'62" E | 2009.05.29 | 30 | yes |  |
| *Taxella eximia* | NGB&CB collection | KU594415 |  | Mal09_181 | Maldives | 5°17'03" S | 73°15'62" E | 2009.05.29 | 30 | yes |  |
| *Taxella eximia* | NGB&CB collection | KU594414 | KY117116 | Mal09_310 | Maldives | 5°34'61" S | 73°08'17" E | 2009.05.31 | 0-40 | yes |  |
| *Taxella eximia* | NGB&CB collection | KU594406 |  | Glo12_065 | Glorieuses | 11°35.80" S | 47°18.52" E | 2012.12.09 | 8-12 | no |  |
| *Taxella eximia* | NGB&CB collection | KU594368 | KY117115 | May07_127 | Mayotte | 12°43'52" S | 45°16'16" E | 2007.12.02 | 4 | no |  |
| *Taxella eximia* | NGB&CB collection |  |  | Eur11_084 | Europa | 22°34'06" S | 40°33'71" E | 2011.11.08 | 10-13 | yes |  |
| *Taxella eximia* | MNHN-IK-16468 |  |  | May06_149 | Mayotte | 12°59'08" S | 45°11'48'' E | 2006.10.26 | 0 to 12 | yes |  |
| *Taxella eximia* | MNHN-IK-16469 |  |  | May06_193 | Mayotte |  |  |  |  | no |  |
| *Taxella eximia* | MNHN-IK-16470 |  |  | May07_018 | Mayotte | 12°52'33'' S | 45°16'52'' E | 2007.11.29 | 0 to 36 | no |  |
| *Taxella eximia* | MNHN-IK-16471 |  |  | May07_069 | Mayotte | 12°51'48'' S | 45°17'27'' E | 2007.11.30 | 0-28 | no |  |
| *Taxella eximia* | MNHN-IK-16472 |  |  | May07_089 | Mayotte | 12°51'48'' S | 45°17'27'' E | 2007.11.30 | 6 to 12 | no |  |
| *Taxella eximia* | MNHN-IK-16473 |  |  | May07_102 | Mayotte | 13°00'21" S | 45°14'44'' E | 2007.12.01 | 9 to 22 | no |  |
| *Taxella eximia* | MNHN-IK-16474 |  |  | May07_110 | Mayotte | 13°00'21" S | 45°14'44'' E | 2007.12.01 | 9 | no |  |
| *Taxella eximia* | MNHN-IK-16475 |  |  | May07_127 | Mayotte |  |  |  |  | no |  |
| *Taxella eximia* | MNHN-IK-16476 |  |  | May07_175 | Mayotte | 12°57'24'' S | 45°31'32'' E | 2007.12.03 | 20 | no |  |
| *Taxella eximia* | MNHN-IK-16477 |  |  | May07_204 | Mayotte | 12°53'50'' S | 45°15'29'' E | 2007.12.05 | 28 | no |  |
| *Taxella eximia* | MNHN-IK-16478 |  |  | May07_234 | Mayotte | 12°53'50'' S | 45°15'29'' E | 2007.12.05 | 10 | no |  |
| *Taxella eximia* | MNHN-IK-16479 | KU594369 | KM587289 | May09_038 | Mayotte | 12°55'05'' S | 44°59'25'' E | 2009.11.29 | 3-15 | no |  |
| *Taxella eximia* | MNHN-IK-16480 | KU594398 | KM587283 | May09_090 | Mayotte | 12°58'85'' S | 44°58'95'' E | 2009.11.30 | 37 | no |  |
| *Taxella eximia* | MNHN-IK-16481 | KU594377 |  | May09_134 | Mayotte | 12°58'85'' S | 44°58'96'' E | 2009.11.30 | 21 | no |  |
| *Taxella eximia* | MNHN-IK-16482 | KU594370 | KM587290 | May09_148 | Mayotte | 12°58'85'' S | 44°58'96'' E | 2009.11.30 | 12 to 17 | no |  |
| *Taxella eximia* | MNHN-IK-16483 | KU594371 |  | May09_160 | Mayotte | 12°58'85'' S | 44°58'96'' E | 2009.11.30 | 8 to 10 | no |  |
| *Taxella gracilicaulis* | MNHN-IK-16484 |  |  | May06_066 | Mayotte | 12°52'10'' S | 45°16'11'' E | 2006.10.24 | 20 to 40 | yes |  |
| *Taxella gracilicaulis* | MNHN-IK-16485 |  |  | May06_067 | Mayotte | 12°52'10'' S | 45°16'11'' E | 2006.10.24 | 20 to 40 | no |  |
| *Taxella gracilicaulis* | MNHN-IK-16486 |  |  | May06_072 | Mayotte | 12°52'10'' S | 45°16'11'' E | 2006.10.24 | 0 to 20 | yes |  |
| *Taxella gracilicaulis* | MNHN-IK-16487 |  |  | May06_094 | Mayotte | 12°52'13'' S | 45°16’38'' E | 2006.10.25 | 40 to 55 | yes |  |
| *Taxella gracilicaulis* | MNHN-IK-16488 |  |  | May06_138 | Mayotte | 12°59'08'' S | 45°11'48'' E | 2006.10.26 | 12 to 15 | no |  |
| *Taxella gracilicaulis* | MNHN-IK-16489 | KU594408 |  | May07_017 | Mayotte | 12°52'33'' S | 45°16'52'' E | 2007.11.29 | 0 to 36 | yes |  |
| *Taxella gracilicaulis* | MNHN-IK-16490 |  |  | May07_157 | Mayotte | 12°57'24'' S | 45°14'44'' E | 2007.12.03 | 40 | no |  |
| *Taxella gracilicaulis* | MNHN-IK-16491 |  |  | May07_168 | Mayotte | 12°57'24'' S | 45°14'44'' E | 2007.12.03 | 30 | yes |  |
| *Taxella gracilicaulis* | MNHN-IK-16492 |  |  | May07_184 | Mayotte | 12°53'50'' S | 45°15'29'' E | 2007.12.05 | 29 | no |  |
| *Taxella gracilicaulis* | MNHN-IK-16493 |  |  | May07_301 | Mayotte | 12°57'24'' S | 45°13'32'' E | 2007.12.07 | 29 | no |  |
| *Taxella gracilicaulis* | MNHN-IK-16494 |  |  | May09_099 | Mayotte | 12°58'85" S | 44°58'96" E | 2009.11.30 | 18 | no |  |
| *Taxella gracilicaulis* | MNHN-IK-16495 |  |  | May09_122 | Mayotte | 12°58'85" S | 44°58'96" E | 2009.11.30 | 6-40 | no |  |
| *Taxella gracilicaulis* | MNHN-IK-16496 |  |  | May09_203 | Mayotte | 12°55'53" S | 44°58'16" E | 2009.12.01 | 16 | no |  |
| *Taxella gracilicaulis* | MNHN-IK-16497 |  |  | May09_518 | Mayotte | 12°55'62" S | 44°58'60" E | 2009.12.07 | 24 | no |  |
| *Taxella gracilicaulis* | MNHN-IK-16498 |  |  | May09_523 | Mayotte | 12°55'62" S | 44°58'60" E | 2009.12.07 | 24 | no |  |
| *Taxella gracilicaulis* | NGB&CB collection | KU594392 | KM587298 | Reu11_153 | Réunion | 21°12'68" S | 55°49'13" E | 2011.11.26 | 15-25 | no |  |
| *Taxella gracilicaulis* | MHNG-INVE-36839 |  | KY117128 | Mad | Madagascar | no data | no data | no data | no data |  |  |
| *Taxella gracilicaulis* | NGB&CB collection | KU594383 | KM587297 | Eur09_052 | Europa | 22°20'29" S | 40°20'08" E | 2009.04.23 | 12-20 | no |  |
| *Taxella gracilicaulis* | NGB&CB collection | KU594383 | KM587299 | Eur09_106 | Europa | 22°20'33" S | 40°19'58" E | 2009.04.24 | 15 | no |  |
| *Taxella gracilicaulis* | NGB&CB collection | KU594383 | KM587300 | Eur09_107 | Europa | 22°20'33" S | 40°19'58" E | 2009.04.24 | 15 | no |  |
| *Taxella gracilicaulis* | NGB&CB collection | KU594382 | KM587301 | Eur11_089 | Europa | 22°34'06" S | 40°33'72" E | 2011.11.08 | 10-13 | yes |  |
| *Taxella gracilicaulis* | NGB&CB collection | KU594384 |  | Eur11_239 | Europa | 22°35'90" S | 40°32'97" E | 2011.11.11 | 12-17 | no |  |
| *Taxella gracilicaulis* | NGB&CB collection | KU594383 |  | Eur11_19new | Europa | no data | no data | 2011.04 | - | yes |  |
| *Taxella gracilicaulis* | NGB&CB collection | KU594383 |  | Eur11_23new | Europa | no data | no data | 2011.04 | - | no |  |
| *Taxella gracilicaulis* | NGB&CB collection | KU594383 |  | Eur11_27new | Europa | no data | no data | 2011.04 | - | no |  |
| *Taxella gracilicaulis* | NGB&CB collection | KU594380 | KM587302 | Jdn09_011 | Juan de Nova | 17°03'44" S | 42°47'04" E | 2009.04.26 | 12-20 | yes |  |
| *Taxella gracilicaulis* | NGB&CB collection | KU594380 | KM587303 | Jdn09_017 | Juan de Nova | 17°03'44" S | 42°47'04" E | 2009.04.26 | 12-20 | yes |  |
| *Taxella gracilicaulis* | NGB&CB collection | KU594385 |  | Jdn09_020 | Juan de Nova | 17°03'44" S | 42°47'04" E | 2009.04.26 | 12-20 | yes |  |
| *Taxella gracilicaulis* | NGB&CB collection | KU594386 |  | Jdn09_124 | Juan de Nova | 17°04'29" S | 42°45'58" E | 2009.04.28 | 10-20 | ? |  |
| *Taxella gracilicaulis* | NGB&CB collection | KU594378 |  | Jdn09_125 | Juan de Nova | 17°04'29" S | 42°45'58" E | 2009.04.28 | 10-20 | ? |  |
| *Taxella gracilicaulis* | NGB&CB collection | KU594379 |  | Jdn09_156 | Juan de Nova | 17°04'29" S | 42°45'58" E | 2009.04.29 | 20 | yes |  |
| *Taxella gracilicaulis* | NGB&CB collection | KU594407 | KY117117 | Mal09_046 | Maldives | 5,00'65" S | 72,87'25" E | 2009.05.27 | 30 | no |  |
| *Taxella gracilicaulis* | NGB&CB collection | KU594407 | KY117118 | Mal09_047 | Maldives | 5,00'65" S | 72,87'25" E | 2009.05.27 | 30 | no |  |
| *Taxella gracilicaulis* | NGB&CB collection | KU594407 | KY117119 | Mal09_401 | Maldives | 5°22'50" S | 73°16'09" E | 2009.06.01 | 20 | yes |  |
| *Taxella hornelli* | NGB&CB collection | KU594423 |  | Mal09_130 | Maldives | 5°14'01" S | 72°92'25" E | 2009.05.28 | 30 | yes |  |
| *Taxella hornelli* | NGB&CB collection | KU594424 | KY117121 | Mal09_423 | Maldives | 5°17'93" S | 72°99'38" E | 2009.06.02 | 35 | no |  |
| *Taxella hornelli* | NGB&CB collection |  | KY117120 | Mal09_424 | Maldives | 5°17'93" S | 72°99'38" E | 2009.06.02 | 35 | no |  |
| *Taxella longicornis* | NGB&CB collection |  |  | Reu | Réunion | no data | no data | 1994.04.07 | 55 | no |  |
| *Taxella longicornis* | NGB&CB collection |  |  | Reu | Réunion | no data | no data | 1994.05.23 | 30 | no |  |
| *Taxella elfica* | MHNG-INVE-36266 |  |  | Mad58 | Madagascar | 12°86' S | 48°59' E | 1958.07.28 | 64 | no |  |
| *Taxella elfica* | MHNG-INVE-36231 |  |  | Mad68 | Madagascar | no data | no data | 1965.06.17 | 60 | yes |  |
| *Gymnangium hians* | MNHN-IK-16431 |  |  | May06_011 | Mayotte | 12°51'34'' S | 45°16’21'' E | 2006.10.23 | 0-25 | yes |  |
| *Gymnangium hians* | MNHN-IK-16432 |  |  | May06_032 | Mayotte | 12°52'10'' S | 45°16’11'' E | 2006.10.24 | 0-60 | yes |  |
| *Gymnangium hians* |  |  |  | May06_033 | Mayotte | 12°52'10'' S | 45°16’11'' E | 2006.10.24 | 0-60 | yes |  |
| *Gymnangium hians* | MNHN-IK-16433 |  |  | May06_034 | Mayotte | 12°52'10'' S | 45°16’11'' E | 2006.10.24 | 0-60 | no |  |
| *Gymnangium hians* | MNHN-IK-16434 |  |  | May06_064 | Mayotte | 12°52'10'' S | 45°16’11'' E | 2006.10.24 | 20 to 40 | yes |  |
| *Gymnangium hians* | MNHN-IK-16435 |  |  | May06_096 | Mayotte | 12°52'13'' S | 45°16'38'' E | 2006.10.25 | 40 to 55 | no |  |
| *Gymnangium hians* | MNHN-IK-16436 |  |  | May06_100 | Mayotte | 12°52'13'' S | 45°16'38'' E | 2006.10.25 | 26-40 | no |  |
| *Gymnangium hians* | MNHN-IK-16437 |  |  | May06_152 | Mayotte | 12°59'08'' S | 45°11'48'' E | 2006.10.26 | 0-12 | no |  |
| *Gymnangium hians* | MNHN-IK-16438 |  |  | May06_153 | Mayotte | 12°59'08'' S | 45°11'48'' E | 2006.10.26 | 0-12 | no |  |
| *Gymnangium hians* | MNHN-IK-16439 |  |  | May06_173 | Mayotte | 12°59'44'' S | 45°15'31'' E | 2006.10.26 | 3 to 8 | no |  |
| *Gymnangium hians* | MNHN-IK-16440 |  |  | May06_196 | Mayotte | 12°59'11'' S | 45°10'53'' E | 2006.10.27 | 5-8 | yes |  |
| *Gymnangium hians* | MNHN-IK-16441 |  |  | May06_197 | Mayotte | 12°59'11'' S | 45°10'53'' E | 2006.10.27 | 5-8 | yes |  |
| *Gymnangium hians* | MNHN-IK-16442 |  |  | May06_199 | Mayotte | 12°58'32'' S | 45°12'18'' E | 2006.10.27 | 0 to 5 | yes |  |
| *Gymnangium hians* | MNHN-IK-16443 |  |  | May06_200 | Mayotte | 12°58'32'' S | 45°12'18'' E | 2006.10.27 | 0 to 5 | no |  |
| *Gymnangium hians* | MNHN-IK-16444 | KU575031 | KY117125 | May07_020 | Mayotte | 12°52'33'' S | 45°16'52'' E | 2007.11.29 | 0 to 36 | yes |  |
| *Gymnangium hians* | MNHN-IK-16445 |  |  | May07_032 | Mayotte | 12°52'33'' S | 45°16'52'' E | 2007.11.29 | 0 to 36 | yes |  |
| *Gymnangium hians* | MNHN-IK-16446 | KU575019 | KY117124 | May07_54 | Mayotte | 12°51'48'' S | 45°17'27'' E | 2007.11.30 | 15-20 | no |  |
| *Gymnangium hians* | MNHN-IK-16447 | KU575031 | KY117126 | May07_55 | Mayotte | - | - | 2007.11.30 | 0 to 3 | yes |  |
| *Gymnangium hians* | MNHN-IK-16448 | KU575050 | KY117123 | May07_61 | Mayotte | 12°51'48'' S | 45°17'27'' E | 2007.11.30 | 0 to 28 | no |  |
| *Gymnangium hians* | MNHN-IK-16449 |  |  | May07_106 | Mayotte | 13°00'21" S | 45°14'44'' E | 2007.12.01 | 9 to 22 | no |  |
| *Gymnangium hians* |  | KU575030 | KY117127 | May07_170 | Mayotte | 13°00'21" S | 45°14'44'' E | 2007.12.03 | 20 | yes |  |
| *Gymnangium hians* | MNHN-IK-16450 |  |  | May07_207 | Mayotte | 12°53'50'' S | 45°15’29'' E | 2007.12.05 | 28 | no |  |
| *Gymnangium hians* | MNHN-IK-16451 |  |  | May07_221 | Mayotte | 12°53'50'' S | 45°15'29'' E | 2007.12.05 | 24 to 26 | yes |  |
| *Gymnangium hians* | MNHN-IK-16452 |  |  | May07_225 | Mayotte | 12°53'50'' S | 45°15'29'' E | 2007.12.05 | 24 to 26 | no |  |
| *Gymnangium hians* | MNHN-IK-16453 |  |  | May07_235 | Mayotte | 12°53'50'' S | 45°15'29'' E | 2007.12.05 | 10 | no |  |
| *Gymnangium hians* | MNHN-IK-16454 |  |  | May07_236 | Mayotte | 12°53'50'' S | 45°15'29'' E | 2007.12.05 | 10 | no |  |
| *Gymnangium hians* | MNHN-IK-16455 |  |  | May07_274 | Mayotte |  |  | 2007.12.06 | 6 to 9 | no |  |
| *Gymnangium hians* | MNHN-IK-16456 |  |  | May07_313 | Mayotte | 12°57'24" S | 45°13'32" E | 2007.12.07 | 30 | no |  |
| *Gymnangium hians* | MNHN-IK-16457 |  |  | May07_351 | Mayotte | 12°51'24" S | 45°15'36" E | 2007.12.08 | 0 to 6 | no |  |
| *Gymnangium hians* | MNHN-IK-16458 |  |  | May07_405 | Mayotte | 12°54'25" S | 45°15'06" E | 2007.12.09 | 19 | no |  |
| *Gymnangium hians* |  |  | KM587305 | May09_010 | Mayotte | 12°55'31" S | 44°57'80" E | 2009.11.29 | 15 to 18 | no |  |
| *Gymnangium hians* | MNHN-IK-16459 |  | KM587313 | May09_104 | Mayotte | 12°58'51" S | 44°58'57" E | 2009.11.30 | 18 | yes |  |
| *Gymnangium hians* | MNHN-IK-16460 |  | KM587310 | May09_147 | Mayotte | 12°58'51" S | 44°58'57" E | 2009.11.30 | 12 to 17 |  |  |
| *Gymnangium hians* | MNHN-IK-16461 |  |  | May09_188 | Mayotte | 12°55'53" S | 44°58'16" E | 2009.12.01 | 29 | yes |  |
| *Gymnangium hians* | MNHN-IK-16462 |  |  | May09_234 | Mayotte | 12°55'32" S | 44°58'10" E | 2009.12.01 | 5 to 6 | no |  |
| *Gymnangium hians* |  |  |  | May09_341 | Mayotte | 12°58'41" S | 44°58'58" E | 2009.12.03 | 27-29 |  |  |
| *Gymnangium hians* | MNHN-IK-16463 |  |  | May09_327 | Mayotte | 12°58'41" S | 44°58'58" E | 2009.12.03 | 33 | no |  |
| *Gymnangium hians* | MNHN-IK-16464 |  |  | May09_426 | Mayotte | 12°40'12" S | 45°03'96" E | 2009.12.04 | 23 | no |  |
| *Gymnangium hians* | MNHN-IK-16465 |  |  | May09_453 | Mayotte | 12°55'54" S | 45°04'58" E | 2009.12.06 | 3 | no |  |
| *Gymnangium hians* | MNHN-IK-16466 |  |  | May09_498 | Mayotte | 12°55'11" S | 45°04'46" E | 2009.12.06 | 0 to 2 | no |  |
| *Gymnangium hians* | MNHN-IK-16467 |  |  | May09_556 | Mayotte | 12°55'37" S | 44°58'36" E | 2009.12.07 | 6 | no |  |
| *Gymnangium hians* | NGB&CB collection | KU575040 | KM587316 | Reu11_325 | Réunion | 21°33'47'' S | 55°50'25'' E | 2011.11.28 | 20-30 | yes |  |
| *Gymnangium hians* | NGB&CB collection | KU575012 |  | Eur09_063 | Europa | 22°20'29" S | 40°20'08" E | 2009.04.23 | 12-20 | yes |  |
| *Gymnangium hians* | NGB&CB collection | KU575012 |  | Eur09_023 | Europa | 22°20'21" S | 40°20'20" E | 2009.04.22 | 12-16 | yes |  |
| *Gymnangium hians* | NGB&CB collection | KU575012 |  | Eur09_024 | Europa | 22°20'21" S | 40°20'20" E | 2009.04.22 | 12-16 | yes |  |
| *Gymnangium hians* | NGB&CB collection | KU575012 |  | Eur09_025 | Europa | 22°20'21" S | 40°20'20" E | 2009.04.22 | 12-16 | yes |  |
| *Gymnangium hians* | NGB&CB collection | KU575012 |  | Eur09_061 | Europa | 22°20'21" S | 40°20'20" E | 2009.04.22 | 12-16 | yes |  |
| *Gymnangium hians* | NGB&CB collection | KU575012 |  | Eur09_062 | Europa | 22°20'29" S | 40°20'08" E | 2009.04.23 | 12-20 | yes |  |
| *Gymnangium hians* | NGB&CB collection |  |  | Eur09_64 | Europa | 22°20'29" S | 40°20'08" E | 2009.04.23 | 12-20 | yes |  |
| *Gymnangium hians* | NGB&CB collection | KU575012 |  | Eur09_066 | Europa | 22°20'29" S | 40°20'08" E | 2009.04.23 | 12-20 | yes |  |
| *Gymnangium hians* | NGB&CB collection | KU575012 |  | Eur09_115 | Europa | 22°20'33" S | 40°19'58" E | 2009.04.24 | 8-19 | yes |  |
| *Gymnangium hians* | NGB&CB collection | KU575012 |  | Eur09_116 | Europa | 22°20'33" S | 40°19'58" E | 2009.04.24 | 8-19 | yes |  |
| *Gymnangium hians* | NGB&CB collection |  | KM587311 | Eur11_090 | Europa | 22°20'44" S | 40°20'23" E | 2011.11.08 | 10-13 |  |  |
| *Gymnangium hians* | NGB&CB collection |  | KM587315 | Eur11_130 | Europa | 22°22'38" S | 40°19'49" E | 2011.11.09 | 11-13 | yes |  |
| *Gymnangium hians* | NGB&CB collection |  |  | Eur11_158 | Europa | 22°24'24" S | 40°22'22" E | 2011.11.09 | 11 | yes |  |
| *Gymnangium hians* | NGB&CB collection |  |  | Eur11_231 | Europa | 22°21'54" S | 40°19'78" E | 2011.11.11 | 12-17 |  |  |
| *Gymnangium hians* | NGB&CB collection |  |  | Gey08_113 | Geyser |  |  | 1905:06:30 |  |  |  |
| *Gymnangium hians* | NGB&CB collection |  | KM587318 | Gey08_085 | Geyser | 12°19'29" S | 46°27'38" E | 2008.04.22 | 8 | no |  |
| *Gymnangium hians* | NGB&CB collection | KU575020 | KM587314 | Gey08_113 | Geyser | 12°21'57" S | 46°27'13" E | 2008.04.23 | 15-20 | no |  |
| *Gymnangium hians* | NGB&CB collection | KU575024 |  | Jdn09_023 | Juan de Nova | 17°03'44" S | 42°47'04" E | 2009.04.26 | 12-20 |  |  |
| *Gymnangium hians* | NGB&CB collection | KU575051 |  | Mal09_141 | Maldives | 5°08'46" S | 72°55'35" E | 2009.05.28 | 0-29 | yes |  |
| *Gymnangium hians* | NGB&CB collection |  |  | Jdn04_92 | Juan de Nova | - | - | 2004.06.26 | - | no |  |
| *Gymnangium hians* | NGB&CB collection |  |  | Jdn11_001 | Juan de Nova | - | - | 2011.04 | 10 | no |  |
| *Gymnangium hians* | NGB&CB collection | KU575038 | KY117122 | Reu13_127,128 | Réunion | - | - | 2013.11.23 | 10-23 | no |  |
| *Gymnangium hians* | MHNG-INVE-86316 | KU512888 |  | KY117129 | Sulawesi, Indonesia | 1°75' S | 125°15' E | 2013.11.29 | 10-15 | no |  |
| *Gymnangium bryani* | NGB&CB collection |  |  | Glo08_022 | Glorieuses | 11°34'53" S | 47°16'52" E | 2008.04.25 | 10 |  |  |
| *Gymnangium bryani* | NGB&CB collection |  |  | Glo03_11 | Glorieuses | - | - | 2003.11. | 10 | yes |  |
| *Gymnangium bryani* | NGB&CB collection |  |  | Glo12_59 | Glorieuses | 11°35'48" S | 47°18'31" E | 2012.12.09 | 8-12 | no |  |
| *Gymnangium bryani* | NGB&CB collection |  |  | Glo12_47 | Glorieuses | 11°35'07" S | 47°20'05" E | 2012.12.09 | 5-15 | yes |  |
| *Gymnangium millardi* | NGB&CB collection |  | KM587275 | Eur11_180 | Europa | 22°38'32" S | 40°38'51" E | 2011.11.10 | 13 | no |  |
| *Gymnangium millardi* | NGB&CB collection |  |  | Mad71_D34 | Madagascar | - | - | 1971.05.24 | no data | no |  |
| *Gymnangium millardi* | SAM-MB-H001923 |  |  |  | North of Durban | 28°13' S | 32°34' E | 1975.01.27 | 48 | yes |  |
| *Gymnangium millardi* | SAM-MB-H003480 |  |  |  |  | 25°57' S | 43°20' E | 1964.08.08 | 42 | no |  |
| *Gymnangium ferlusi* | NGB&CB collection |  |  | Mad69_34 | Madagascar | - | - | 1969.05.22 | 34 | yes |  |
| *Gymnangium ferlusi* | MHNG-INVE-36222 |  |  | Mad | Madagascar, Manafiaty | 24°46' S | 47°11' E | no data | 60 | no |  |
| *Gymnangium ferlusi* | MHNG-INVE-36273 |  |  | Mad | Madagascar | 25°02' S | 47°00' E | 1958.10.19 | 50 | no |  |
| *Gymnangium expansum* | MHNG-INVE-69623 | KU512884 | KY117129 |  | Okinawa, Japan | 26°14'20" S | 126°49'05" E | 2009.11.20 | 141-165 | no |  |
| *Gymnangium insigne* |  | KM587319 | KM587319 | HYTH12 | Tahiti |  |  |  |  |  |  |
| *Gymnangium insigne* |  |  | KM587320 | HYTH63 | Tahiti |  |  |  |  |  |  |
| *Gymnangium montagui* | MHNG-INVE-89694 |  |  |  | France, Brest | 48°19'43" N | 4°27'27" W | 2014.09.08 | 12 |  |  |
| *Gymnangium montagui** |  | JN560075 | KY117130 |  | Portugal, Berlengas | 39°25'24" S | 9°29'54" W |  | 25-35 |  |  |
| *Gymnangium montagui** |  | JN560076 |  |  | Portugal, Sines, Porto Covo | 37°51'19" S | 8°48'21" W |  | 15-20 |  |  |
| *Gymnangium montagui** |  | JN560077 |  |  | Portugal, Berlengas, Estelas | 39°25'02" S | 9°32'28" W |  | 15-26 |  |  |
| *Gymnangium montagui** |  | JN560078 |  |  | Portugal, Sagres | 36°59'30" S | 8°56'32" W |  | 17-22 |  |  |

*after Moura CJ, Cunha MR, Porteiro FM, Rogers AD. A molecular phylogenetic appraisal of the systematics of the Aglaopheniidae (Cnidaria : Hydrozoa , Leptothecata) from the north-east Atlantic and west Mediterranean. Zool J Linn Soc. 2012; 164: 717-727.
