# Supplementary material for: Modern alongside traditional taxonomy—Integrative systematics of the genera Gymnangium Hincks, 1874 and Taxella Allman, 1874 (Hydrozoa, Aglaopheniidae)
Source: PLoS One. 2017 Apr 19;12(4):e0174244. doi: 10.1371/journal.pone.0174244 (PMC5396908; doi:10.1371/journal.pone.0174244)
Supplement: S2 Appendix — PC1, PC2 and PC3 indicate the respective weights of each parameter in the PCA. (DOCX) [file pone.0174244.s002.docx]

Appendix S2. Parameters used in the PCA analysis. PC1, PC2 and PC3 indicate the respective weights of each parameter in the PCA.

**Variable PC1 PC2 PC3**

1 – stem ramification (yes, no) -0,256 0,034 -0,042

2 – colony flexuous (yes, no) -0,179 0,105 -0,112

3 – colony 2-dimensional (yes, no) 0,029 -0,156 0,127

4 – stem polysiphonic (yes, no) 0,257 0,030 0,029

5 – presence of axial tube with short hydrocladia (yes, no) 0,257 0,030 0,029

6 – branching pattern (absent, irregular, alternate, opposite) -0,253 0,011 -0,033

7 – presence of hinge-joint (yes, no) -0,160 0,048 -0,026

8 – maximum number of hydrocladial article 0,226 0,005 0,146

9 – hydrocladia_maximum length 0,234 -0,026 0,067

10 – hydrocladia_internode length 0,114 0,390 -0,221

11 – presence of pseudophylactocarp (yes, no) -0,052 0,030 -0,006

12 – hydrotheca_shape (cup-shaped, tubular) 0,256 -0,034 0,042

13 – hydrotheca_mean depth 0,095 0,617 -0,216

14 – hydrotheca_opening width 0,249 0,063 0,050

15 – hydrotheca_abcauline intrathecal septum (yes, no) 0,256 -0,034 0,042

16 – hydrotheca_abcauline thickening below aperture (yes, no) -0,227 -0,022 0,178

17 – hydrotheca_number of lateral teeth 0,253 0,043 -0,032

18 – hydrotheca_distance between tip of median nematotheca

and abcauline tip of hydrotheca -0,103 0,620 0,200

19 – lateral nematotheca_length 0,039 0,088 0,353

20 – lateral nematotheca_position (above, level, below the level

of hydrotheca, variable) 0,194 -0,013 0,116

21 – median nematotheca_length 0,110 0,120 0,427

22 – median nematotheca_length of free part 0,071 0,038 0,542

23 – median nematotheca_number of apertures -0,233 0,060 0,319

24 – median nematotheca_opening (circular, pointed) 0,245 -0,047 -0,158

25 – median nematotheca_shape (tubular, gut) 0,245 -0,047 -0,158
