# Supplementary material for: Modern alongside traditional taxonomy—Integrative systematics of the genera Gymnangium Hincks, 1874 and Taxella Allman, 1874 (Hydrozoa, Aglaopheniidae)
Source: PLoS One. 2017 Apr 19;12(4):e0174244. doi: 10.1371/journal.pone.0174244 (PMC5396908; doi:10.1371/journal.pone.0174244)
Supplement: S4 Appendix — (DOC) [file pone.0174244.s004.doc]

**S4 Appendix. Measurements of species of genus *Taxella.***

| ***Taxella*** | ***T. eximia*** | ***T. gracilicaulis*** | ***T. hornelli*** | ***T.  longicornis*** | ***T. elfica* sp. nov.** |
| --- | --- | --- | --- | --- | --- |
| **Character** | **Mean (± SD) [μm]** | **Mean (±SD) [μm]** | **Mean (μm)** | **Range [μm]** | **Range [μm]** |
| Branchlet internode length | - | - | 394^ | - | - |
| Hinge-joint longer length | - | 1265 (± 239) | 1080 - 1209^ | 878 - 1075 | - |
| Hydrocladium internode length | 263 (± 26) | 343 (± 60) | 311 - 395^ | 211 - 249 | 307 - 400 |
| Hydrotheca depth | 217 (± 13.9) | 268 (± 25) | 289 - 313^ | 232 - 273 | 313 - 370 |
| Diameter at mouth, frontal view | 129 (± 5.7) | 132 (± 6) | 128^ | 104 - 122 | 144 - 170 |
| Median nematotheca, total abcauline length | 193 (± 8.9) | 214 (± 12) | 231 - 249^ | 257 - 306 | 304 - 403 |
| Median nematotheca, length of free part | 51 (± 4.5) | 53 (± 6) | 49 - 55^ | 90 - 135 | 52 - 67 |
| Distance between tip of median nematotheca and abcauline tip of hydrotheca | 69 (± 11.4) | 117 (± 23) | 96 - 103^ | 42 - 112 | 111 - 196 |
| Lateral nematotheca length | 107 (± 6.7) | 115 (± 6) | 109 - 111^ | 143 - 172 | 83 - 122 |
| Gonotheca length | 358 (± 54) | 399 (± 44) | 439 - 511 * | - | 508 - 667 |
| Gonotheca width | 284 (± 40) | 288 (± 43) | 288 - 361 * | - | 352 - 438 |

^ range of means of two specimens

* range

**S4 Appendix. Measurements of species of genus *Gymnangium.***

| ***Gymnangium*** | ***G. hians***  **form with 3 teeth** | ***G. hians***  **form with 2 teeth** | ***G. bryani*** | ***G. millardi* sp. nov.** | ***G. ferlusi*** |
| --- | --- | --- | --- | --- | --- |
| **Character** | **Mean (± SD) [μm]** | **Mean (±SD) [μm]** | **Mean (μm)** | **Range [μm]** | **Mean (range)** [**μm]*** |
| Hydrocladium internode length | 357 (± 18) | 318 (± 16) | 389 (± 68) | 172 - 236 | 298 (237 - 353) |
| Hydrotheca depth | 290 (± 14) | 243 (± 7) | 263 (± 10) | 182 - 199 | 232 (215 - 247) |
| Diameter at mouth, frontal view | 283 (± 12) | 232 (± 15) | 205 (± 19) | 189 - 224 | 290 (285 - 296) |
| Median nematotheca, total abcauline length | 254 (± 19) | 217 (± 9) | 219 (± 48) | 284 - 340 | 408 (399 - 429) |
| Median nematotheca, length of free part | 62 (± 9) | 54 (± 4) | 47 (± 7) | 167 - 187 | 169 (138 - 238) |
| Distance between tip of median nematotheca and abcauline tip of hydrotheca | - | - | 94 (± 45) | 140 - 203 | - |
| Lateral nematotheca length | 118 (± 9) | 107 (± 5) | 74 (± 14) | 106 - 115 | 173 (149 - 198) |
| Gonotheca length | 690 (± 60) | - | 594 (± 26) | - | 386 |
| Gonotheca width | 551 (± 28) | - | 388 (± 18) | - | 465 |

* no SD given because one specimen measured
